# UAP56 is a conserved crucial component of a divergent mRNA export pathway in *Toxoplasma gondii*

**DOI:** 10.1111/mmi.13485

**Published:** 2016-09-14

**Authors:** Mariana Serpeloni, Elena Jiménez‐Ruiz, Newton Medeiros Vidal, Constanze Kroeber, Nicole Andenmatten, Leandro Lemgruber, Patricia Mörking, Gurman S. Pall, Markus Meissner, Andréa R. Ávila

**Affiliations:** ^1^ Instituto Carlos Chagas, FIOCRUZCuritibaBrazil; ^2^ Departamento de Biologia Celular e MolecularUniversidade Federal do ParanáCuritibaBrazil; ^3^ College of Medical, Veterinary and Life Sciences, Institute of Infection, Immunity & Inflammation, Wellcome Trust Centre for Molecular Parasitology, University of GlasgowUK; ^4^ National Center for Biotechnology Information, National Library of Medicine, National Institutes of HealthBethesdaMDUSA

## Abstract

Nucleo‐cytoplasmic RNA export is an essential post‐transcriptional step to control gene expression in eukaryotic cells and is poorly understood in apicomplexan parasites. With the exception of UAP56, a component of TREX (Transcription Export) complex, other components of mRNA export machinery are not well conserved in divergent supergroups. Here, we use *Toxoplasma gondii* as a model system to functionally characterize TgUAP56 and its potential interaction factors. We demonstrate that TgUAP56 is crucial for mRNA export and that functional interference leads to significant accumulation of mRNA in the nucleus. It was necessary to employ bioinformatics and phylogenetic analysis to identify orthologs related to mRNA export, which show a remarkable low level of conservation in *T. gondii*. We adapted a conditional Cas9/CRISPR system to carry out a genetic screen to verify if these factors were involved in mRNA export in *T. gondii*. Only the disruption of TgRRM_1330 caused accumulation of mRNA in the nucleus as found with TgUAP56. This protein is potentially a divergent partner of TgUAP56, and provides insight into a divergent mRNA export pathway in apicomplexans.

## Introduction

Nucleo‐cytoplasmic RNA export is an essential post‐transcriptional pathway to control gene expression in eukaryotic cells. In Metazoan and Fungi, the nuclear export of most RNA species (such as microRNAs, ribosomal rRNAs, small nuclear RNAs and transfer RNAs) requires specific exportins and the small GTPase Ran. In contrast, nuclear export of bulk messenger RNAs (mRNAs) is Ran‐exportin independent (Cullen, 2003; Kohler and Hurt, 2007). The mRNA export machinery is tightly coupled to mRNA splicing and includes different sets of mRNA binding proteins and nucleoporins (Rodriguez‐Navarro *et al*., 2004; Kohler and Hurt, 2007; Wolyniak and Cole, 2008). In general proteins of THO complex associate with mRNA and recruit processing and export factors resulting in the formation of the Transcription/Export (TREX) complex (Strasser *et al*., 2002). TREX complex interacts with the spliceosome and mature mRNAs are exported through the nuclear pore complex (NPC) by binding to the heterodimeric receptor Mex67:Mtr2/TAP:p15, in an Ran‐independent manner (reviewed by (Katahira, 2015; Wickramasinghe and Laskey, 2015)). The mRNAs are then released for translation into the cytoplasm by ATP‐dependent helicase Dbp5/DDX19 (Kohler and Hurt, 2007; Nino *et al*., 2013).

While mRNA export is well understood in opisthokonts, in the case of early divergent supergroups, such as Chromalveolata and Excavata, proteins involved in this pathway are not conserved. The only exception is Sub2 (UAP56), a DEAD‐box helicase and member of TREX complex (Serpeloni *et al*., 2011b). While a role of UAP56 in mRNA processing has been suggested for the apicomplexa *Plasmodium falciparum* (Shankar *et al*., 2008), its role in mRNA export remains unknown. Using the apicomplexan *Toxoplasma gondii* as model, we show that knocking‐out TgUAP56 led to a significant block of mRNA export, suggesting the presence of a specific mRNA export route akin to other eukaryotes. However, the identification of interaction partners of this crucial protein was not straightforward using standard search analysis due to low conservation of the components. Instead, we employed bioinformatics and phylogenetic analysis to identify *T. gondii* orthologs of factors that have been described as essential for mRNA export in opisthokonts. We identified some orthologs and discovered that these proteins sequences are very divergent in comparison with orthologs from other species. We predicted we would find the main mRNA export receptor in eukaryotes, Mex67, however our bioinformatics and phylogeny analysis failed to reveal its ortholog in *T. gondii*. Therefore, it may be possible that in apicomplexans, unlike in opisthokonts, mRNA is exported by a non‐conserved export pathway in an Exportin/Ran‐dependent manner. To dissect the factors involved in mRNA export in *T. gondii*, and to discriminate between Ran‐dependent and ‐independent routes, we used reverse genetic strategies to interfere with specific components of both pathways.

As part of these strategies we developed a conditional Cas9/CRISPR in *T. gondii* to target a subset of potential candidates to identify factors involved in mRNA export. Functional interference with GTPAse Ran and the exportin CRM1 does not lead to bulk mRNA export defects, suggesting that mRNA export is Exportin/Ran‐independent in *T. gondii*, consistent with other eukaryotes. In addition other orthologs analyzed in this work, including Exportin/Ran‐independent factors, were not crucial for mRNA export pointing to the presence of potential unique components for mRNA export in *T. gondii*. Functional interference with TgRRM_1330, which interacts with TgUAP56 and seems to be a divergent ortholog of Yra1/Aly, led to a phenocopy of TgUAP56 KO. Here, we discuss that TgUAP56 and the divergent TgYra1 may be the first components of a divergent mRNA export pathway operating in apicomplexan parasites, and how these can be used empirically to identify other components that to date could not be identified through *in silico* sequence‐based homology screens.

## Results

### UAP56 is crucial for mRNA export in *T. gondii*

We have previously shown that most components central to the Ran‐independent mRNA export pathway are not conserved in organisms that diverged early during evolution (Serpeloni *et al*., 2011b). One exception that is conserved in all eukaryotes is the DEAD‐box helicase UAP56, which is also involved in splicing of pre‐mRNAs (Fleckner *et al*., 1997; Jensen *et al*., 2001; Libri *et al*., 2001; Strasser and Hurt, 2001; Thakurta *et al*., 2007). To reconstruct the phylogeny of UAP56 orthologs sequences across eukaryotes, we surveyed 43 representative species of different eukaryotic groups. The data presented in Supporting Information Figure S1 show that UAP56 protein phylogeny resembles the eukaryotic species phylogeny. The orthologs protein of UAP56 in *T. gondii*, TgUAP56, is annotated as a DEAD‐box polypeptide DDX39 (ID TGME49_216860 at ToxoDB, GI 237843393 at NCBI) and is 78.6% and 72.8% similar to orthologs in human cells (Hsa_UAP56) and yeast (Sce_Sub2) respectively. Multiple sequence alignments of TgUAP56 and representative sequences from other eukaryotic groups demonstrate high sequence conservation along the entire protein including two RNA helicase domains.

**Figure 1:**
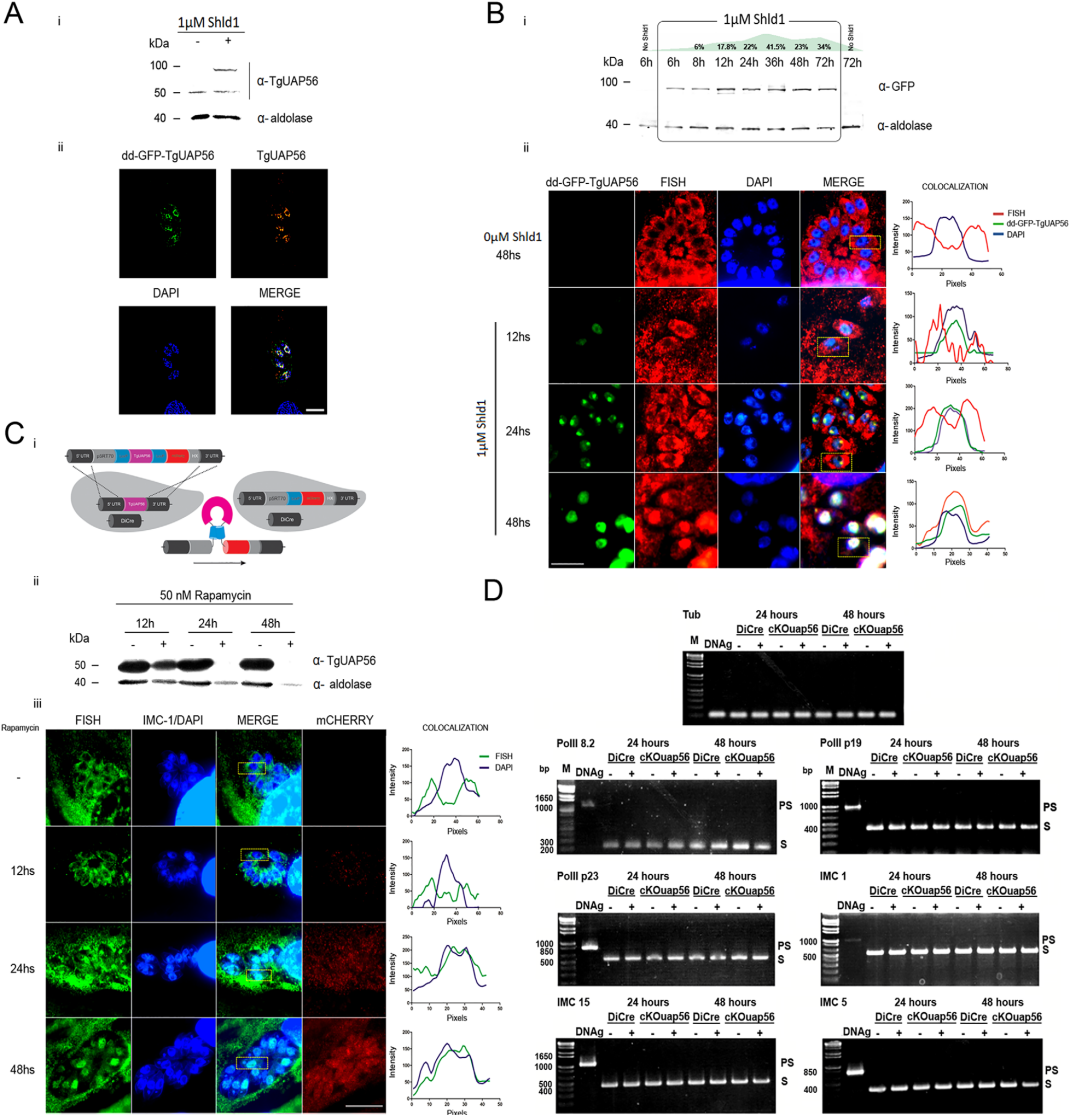
Localization and functional analysis of TgUAP56. A. Analysis of dd‐GFP‐TgUAP56 expression. i) In non‐induced parasites (‐Shld1), only the endogenous protein (TgUAP56) is detected by Western blot using polyclonal antibody against Tryp‐Sub2 (Serpeloni *et al*., 2011a), renamed here as α‐TgUAP56. Both TgUAP56 and dd‐GFP‐TgUAP56 are detected by this antibody after incubation with 1 µM of Shld1 for 6 h. Aldolase: Loading control. ii) In the immunofluorescence assay dd‐GFP‐TgUAP56 is nuclear and colocalizes with endogenous TgUAP56 protein, in red. Nuclear and apicoplast DNA staining with DAPI: in blue. Scale bar: 5 µm. B. Analysis of mRNA distribution after dd‐GFP‐TgUAP56 overexpression with 1 µM Shld1. i) Western blot to analyze induction of dd‐GFP‐TgUAP56. The protein was detected with anti‐GFP. Aldolase was used as loading control. dd‐GFP‐TgUAP56 was detectable after 6 h of incubation with 1 µM of Shld1. The numbers above the Western blot indicate the relative expression levels of dd‐GFP‐TgUAP56 at each indicated time point, normalized to the loading control aldolase using ImageJ software with the densitometry plugin (Version 1.6, National Institutes of Health, Bethesda, MD). ii) To check mRNA distribution, poly(A)^+^ mRNAs were detected by fluorescent *in situ* hybridization (FISH) using oligodT‐Alexa594 as probe: in Red. Nuclear and apicoplast DNA staining with DAPI: in blue. dd‐GFP‐TgUAP56: in green. In the right: quantification of signals of immunofluorescence and DAPI in selected parasites, in yellow box. Scale bar: 10 µm. C. Analysis of mRNA distribution after knockout of *uap56* by gene‐swap strategy based on Di‐Cre system in *T. gondii*. i) Endogenous *uap56* gene is replaced with *mcherry* by Dicre after incubation with 50 nM of rapamycin. ii) *uap56* knockout was analyzed in cKOuap56 strain western blot after incubation with 50 nM of rapamycin at different times. ‐, not induced; +, induced. Aldolase: Loading control. iii) mRNA distribution was analyzed in cKOuap56 strain after incubation with rapamycin at different times by fluorescent *in situ* hybridization (FISH) using oligodT‐Alexa488 as probe, in green. Nuclear and apicoplast DNA was staining with DAPI: in blue. In the right: quantification of signals of immunofluorescence and DAPI in selected parasites. Scale bar: 10 µm D. PCR analysis of mRNA splicing for selected genes. Total RNA was purified from DiCre strain and cKOuap56 strain both incubated with 50 nM of rapamycin, as indicated. −, not induced; +, induced. The total RNA was reverse transcribed and PCR amplified using primers that span an intron. PCR of gDNA was included as a reference to distinguish between properly spliced (S) and pre‐spliced (PS) forms of each gene. In the top: Tubulin, used as loading control (Dalmasso *et al*., 2009). In the bottom: PCR analysis of mRNA splicing for selected genes: RNA polymerase II *p8.2* subunit (TGME49_217560), RNA polymerase II *p19* subunit (TGME49_271300), RNA polymerase II *p23* subunit (TGME49_240590), *imc1* (TGME49_231640), *imc15* (TGME49_275670), *imc5* (TGME49_224530), and transcription factor *iid* (TGME49_258680). gDNA: genomic DNA from non‐induced parasites of cKOuap56 strain. Expected sizes for pre‐mRNA (Pre‐spliced) and mRNA (spliced) are shown for each selected gene.

To characterize the function of TgUAP56, we generated transgenic parasites expressing TgUAP56 N‐terminally fused to ddFKBP‐GFP (Breinich *et al*., 2009) (dd‐GFP‐TgUAP56). The ddFKBP domain allows conditional stabilization of the fusion protein in the presence of the ligand Shield‐1 (Shld1) (Banaszynski *et al*., 2006). We verified that incubation of transgenic parasites with 1 µM Shld1 results in stabilization of dd‐GFP‐TgUAP56. Furthermore, dd‐GFP‐TgUAP56 co‐localizes with endogenous TgUAP56 in the nucleus, as shown by immunofluorescence analysis using a polyclonal antibody raised against the trypanosome ortholog (Serpeloni *et al*., 2011a), which specifically detects TgUAP56 (Fig. 1A‐i, ii). dd‐GFP‐TgUAP56 stabilization is very efficient and the protein can be detected as early as 6 h after addition of 1 µM Shld1, reaching a peak at 36 h (Fig. 1B‐i). We found that overexpression of dd‐GFP‐TgUAP56 efficiently interferes with parasite growth indicating functional interference with endogenous TgUAP56 and an essential role for this protein (Supporting Information Fig. S2A). We speculated that block of parasite growth is caused by interference with mRNA export. Indeed, the incubation of parasites for 48 h in presence of Shld1 caused an obvious nuclear accumulation of mRNA that co‐localizes with dd‐GFP‐TgUAP56 (Fig. 1B‐ii).

To exclude that the phenotype observed after overexpression of dd‐GFP‐TgUAP56 is due to a non‐specific interference with the mRNA export pathways, we used an inducible gene‐swap strategy (Andenmatten *et al*., 2013) to generate a conditional knockout for *uap56* (cKOuap56). This strategy is based on site‐specific recombination by DiCre, which catalyzes excision of DNA flanked by *loxP* sites after induction with rapamycin (Andenmatten *et al*., 2013; Bargieri *et al*., 2013). Parental DiCre strain was transfected with cKOuap56 plasmid to generate a stable cell line for conditional knockout of *uap56* gene (cKOuap56 strain). Upon induction, excision of *uap56* results in replacement of the reporter gene *mcherry* under control of the endogenous promoter and hence knockout parasites can be identified based on red fluorescence (Fig. 1C‐i). We confirmed correct homologous recombination of the *uap56‐LoxP* cassette in the original *uap56* locus by gDNA PCR analysis (Supporting Information Fig. S2B‐i, primers set 1 and 2, A and C comparison). *uap56* gene excision was confirmed at genomic level by gDNA PCR analysis after 24 h induction with rapamycin (Supporting Information Fig. S2B‐ii, primers set 1 and 2, A and B comparison). Real‐time quantitative PCR and immunoblot assays were performed to analyze *uap56* mRNA and TgUAP56 protein levels, respectively, at specific time points after rapamycin induction. Non‐induced and induced parasites of parental DiCre strain have normal levels of *uap56* mRNA in the presence of rapamycin at all time points tested (Supporting Information Fig. S2C‐i). In the case of induced parasites of cKOUAP56 strain, *uap56* mRNA levels decreased drastically 48 h after addition of rapamycin (Supporting Information Fig. S2C‐ii). In good agreement, the levels of TgUAP56 protein significantly decreased after 24 h of rapamycin incubation and after 48 h it is virtually undetectable (Fig. 1C‐ii). The nuclear accumulation of poly(A)^+^ RNA is observed after 48 h when levels of TgUAP56 are undetectable (Fig. 1C‐iii). The incubation of parasites in presence of 50 nM rapamycin has no toxic effect on parasites as demonstrated previously (Andenmatten *et al*., 2013). In our case, the treatment of rapamycin itself in the parental DiCre strain did not affect the expression levels of TgUAP56 (Supporting Information Fig. S2E‐i) or the export of mRNAs (Supporting Information Fig. S2E‐ii). In addition, the detection of *mcherry* expression after rapamycin incubation confirmed that *uap56*‐*loxP* was excised successfully (Fig. 1C‐iii). Furthermore, we confirmed that TgUAP56 is essential, since *uap56* excision caused a lethal phenotype (Supporting Information Fig. S2D). Importantly, a similar mRNA accumulation in the nucleus was observed after dd‐GFP‐TgUAP56 stabilization, demonstrating that this phenotype is specific and therefore an essential role for TgUAP56 in mRNA export.

Next we surveyed a selection of genes by a semi‐quantitative PCR analysis developed by Suvorova *et al*. (2013), using primers spanning an intron as listed in Supporting Information Table S2 to assess if TgUAP56 plays an important role in mRNA‐splicing. Parasites of parental DiCre and cKOuap56 strains were incubated with 50 nM of rapamycin for 24 and 48 h before extraction of total RNA. gDNA extracted from cKOuap56 strain was used as a reference for intron‐containing pre‐spliced mRNAs. PCR data did not show accumulation of pre‐mRNA for any of the genes analyzed indicating that *uap56* knockout does not affect mRNA splicing (Fig. 1D).

### Bioinformatic and phylogenetic analysis provide the identification of ortholog proteins in *T. gondii*

Since TgUAP56 is related to mRNA export in *T. gondii*, we decided to systematically probe the genome of the parasite to identify proteins that are potentially related to TgTREX (*T. gondii* TREX complex) and downstream events. Our first approach was based on protein sequence search and phylogenetic reconstruction. The general criteria to choose the candidates were: (a) evidence of Sub2/UAP56 interaction partners and/or (b) they are essential for mRNA export in humans or yeast.

Sub2/UAP56 and Yra1/Aly are known partners that are part of TREX complex in opisthokonts and are loaded onto mRNAs in a splicing‐dependent manner (for reviews, see (Reed and Hurt, 2002; Custodio *et al*., 2004; Masuda *et al*., 2005)). TREX can recruit the RNA export receptor Mex67:Mtr2 to spliced mRNAs (Gilbert and Guthrie, 2004; Hurt *et al*., 2004; Iglesias *et al*., 2010; Hackmann *et al*., 2014) and it is known that TAP:p15 can be targeted to spliced mRNPs directly to a spliceosome U2AF35 subunit and this interaction is conserved across metazoan species (Zolotukhin *et al*., 2002). In addition to these RNA binding proteins, Npl3 and Gbp2, the latter associated with TREX complex, can recruit mRNA export receptor Mex67:Mtr2 to mRNAs also and are crucial for formation of export‐competent mRNP (Gilbert and Guthrie, 2004; Hurt et al., 2004; Iglesias et al., 2010; Hackmann et al., 2014). Besides, mutants of *npl3* show strong mRNA‐export defects (Lee *et al*., 1996; Krebber *et al*., 1999; Strasser and Hurt, 2000; Gilbert *et al*., 2001; Gilbert and Guthrie, 2004; Gwizdek *et al*., 2006) and overexpression of Gbp2 is toxic and causes a nuclear retention of bulk poly(A)^+^ RNA (Windgassen and Krebber, 2003).

Our approaches allowed the identification of orthologs of most proteins cited above in *T. gondii*, although low sequence conservation is observed in Kinetoplastida and Apicomplexa (Table 1, Supporting Information Figs S4–7). However, this approach did not allow the identification of a *T. gondii* ortholog of Mex67, the specific receptor of mRNA export in yeast. Based on the fact that GTPase Ran and CRM1 are required for the export of proteins and a subset of mRNAs in some eukaryotes (Cullen, 2003; Wickramasinghe and Laskey, 2015, reviewed by (Delaleau and Borden, 2015), we used the *T. gondii* orthologs to assess if mRNA export was Ran‐dependent in *T. gondii*. Exportin CRM1 and its cofactor GTPase Ran are highly conserved throughout eukaryotic phylogeny (Supporting Information Figs S8 and 9) (Serpeloni *et al*., 2011b). To analyze the potential role of the candidates in mRNA export we took advantage of the conditional gene disruption by ddCas9, as described below.

**Table 1:** mRNA export candidates in *T. gondii* based on sequence and phylogenetic analysis.

*S. cerevisiae*	Metazoans	ID	NCBI – description	References	Length (aa)	PFAM – domains	ToxoDB – description	ID	Length (aa)	PFAM – domains
Yra1p	Aly/REF	GI: 6320589	Nuclear polyadenylated RNA‐binding protein; required for export of poly(A)^+^ mRNA from the nucleus. Is deposited onto mRNAs through its interaction partner Sub2/UAP56 and couple mRNA export with 3′ end processing via its interactions with Mex67p	Lou *et al*. (2001), Zhou (2000), Masuda *et al*. (2005), Meinel *et al*. (2013), Ma *et al*. (2013), and Johnson *et al*. (2011)	226	RRM_Aly_REF	RNA recognition motif‐containing protein	TGME49_291330 (TgRRM_1330)	228	RRM_1
Npl3		PF10_0217 (serine/arginine‐rich splicing factor 4 (SRSF4))	In yeast: RS containing shuttling RNA‐binding‐protein recruited to the mRNAs co‐transcriptionally early by RNA polymerase II and is required for pre‐mRNA splicing. Phosphorylation is essential for efficient competent mRNP export.	Tuteja and Mehta (2010)	538	RRM1_RRM1	Splicing factor SF2 (SF2)	TGME49_319530 (TgSF2_9530)	512	RRM_1/RRM_1
Gbp2		PF10_0068 (RNA‐binding protein, putative)	In yeast: poly(A)^+^ RNA‐binding protein recruited to the mRNAs co‐transcriptionally via THO complex involved in mRNA surveillance and nuclear mRNA quality control.	Tuteja and Mehta (2010)	246	RRM1_RRM1	RNA recognition motif‐containing protein	TGME49_262620 (TgRRM_2620)	293	RRM_1/RRM_1
	U2AF35	GI: 68800128	RNA recognition motif in U2 small nuclear ribonucleoprotein auxiliary factor U2AF 35 kDa subunit, that directly binds to TAP, and this interaction is conserved across metazoan species	Wu *et al*. (1999) and Zolotukhin *et al*. (2002)	240 aa	zf‐CCCH/RRM_5/zf‐CCCH	U2 snRNP auxiliary factor, putative	TGME49_236910 (TgU2_6910)	254	zf‐CCCH/zf‐CCCH/RRM_5
CRM1	Xpo1	GI: 398366207	Major karyopherin/exportin involved in export of proteins, snRNAs, rRNAs, viral RNAs and a subset of endogenous mRNAs	Hammell *et al*. (2002), Cullen (2003), Koyamae Matsuura (2010), Sun *et al*. (2013), and Wickramasinghe and Laskey (2015)	1084	CRM1 _C/Xpo1/IBN_N	Exportin 1, putative	TGME49_249530 (TgCRM1)	1125	CRM1_C/Xpo1/IBN_N
Ran GTPase GSP1	Ran	GI: 6323324	Ran GTPase; GTP binding protein involved in the maintenance of nuclear organization, RNA processing and transport	Cullen (2003) and Wickramasinghe and Laskey (2015)	219	Ras	GTP‐binding nuclear protein ran/tc4	TGME49_248340 (TgRan)	229	Ras

### Conditional Cas9 expression allows identification of mRNA export mutants

**Figure 2:**
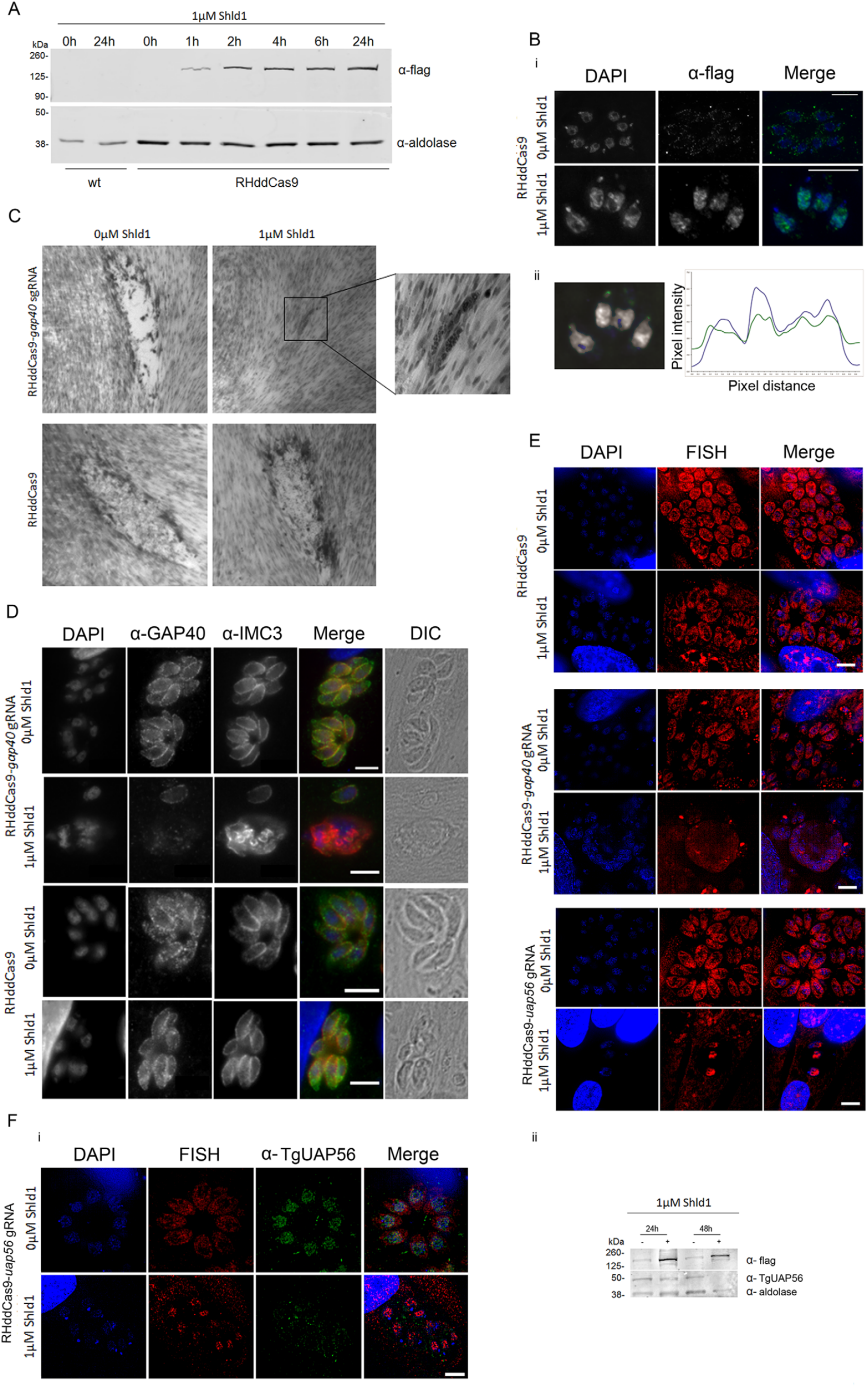
Establishment of a conditional Cas9 (ddCas9). A. Western blot showing ddCas9‐FLAG overexpression. Parasites were induced for indicated times with 1µM of Shld1 prior protein extraction. RH‐Δhxgprt strain was used as control (first and second lanes ‐ *wild type‐*wt) Aldolase: Loading control. B. i) Nuclear localization of Cas9 in RHddCas9 parasites after addition of 1 µM Shld1 for 24 h. Nuclear and apicoplast DNA staining with DAPI: in blue. Scale bar: 10µm. ii) Co‐localization map, showing in grey the areas where there was co‐localization with an M2 of 0.8. Graph represents the areas where there was a signal for green (green line) and areas where there was signal for blue (blue line). *y*‐Axis: intensity level; *x*‐axis: distance in microns. C. Plaque assays for parental RHddCas9 and RHddCas9‐*gap40* gRNA strains. Both parasite strains were grown on human foreskin fibroblasts, in the presence or absence of 1 µM of Shld1, as indicated, for 108 h. −, not induced; +, induced. Scale bar: 500 µm. D. Immunofluorescence assay of parental RHddCas9 and RHddCas9‐*gap40* sgRNA strains in the presence or absence of Shld1. Collapsing vacuoles with a significant reduction of GAP40 signal were only observed in induced parasites that expressed the specific sgRNA against *gap40*. Scale bar: 8 µm. E. mRNA distribution was analyzed in RHddCas9, RHddCas9‐*gap40* gRNA and RHddCas9‐*uap56* gRNA strains after incubation for 4 h with 1 µM Shld1 and further incubation for 48 h in media. The analysis was performed by fluorescent *in situ* hybridization (FISH) using oligodT‐Alexa594 as probe, in red. Nuclear and apicoplast DNA was staining with DAPI: in blue. Scale bar: 5 µm. F. mRNA distribution and TgUAP56 protein presence analysis in RHddCas9‐*uap56* gRNA strain after incubation for 4 h with 1 µM Shld1 and further incubation for 48 h in media. i) The analysis was performed by immunofluorescence assay, α‐TgUAP56 in green, combined with fluorescent *in situ* hybridization (FISH) using oligodT‐Alexa594 as probe, in red. mRNA export blocking was observed in absence of TgUAP56 in induced parasites that expressed the specific sgRNA against *uap56*. Nuclear and apicoplast DNA was staining with DAPI: in blue. Scale bar: 5µm. ii) Western blot to analyse TgUAP56 protein levels after 1 µM Shld1 incubation for 24 and 48 h. The protein was detected with anti‐TgUAP56. Aldolase was used as loading control. ddCas9‐FLAG was detected using antibody α‐Flag. −, not induced; +, induced.

The recent adaptation of CRISPR/Cas9 in *T. gondii* parasites is a powerful technology to generate direct knockouts for non‐essential genes in a rapid and reliable manner (Shen *et al*., 2014; Sidik *et al*., 2014). Transient expression of Cas9 might be helpful to rapidly identify certain phenotypes (Harding *et al*., 2016), but constitutive expression of Cas9 might lead to artifacts. To overcome this limitation we designed a conditional nuclear Cas9 fused to ddFKBP to allow precise regulation of Cas9 expression levels (Supporting Information Fig. S3A). ddCas9 is detectable as early as 1 h after addition of Shld1 (Fig. 2A) and localizes mainly to a defined region within the nucleus in all parasites. Longer stabilization leads to Cas9 localization throughout the nucleus of the parasite (Fig. 2B, Supporting Information Fig. S3B). Importantly, while short incubation times, up to 4 h, with Shld1 did not significantly affect parasite viability, longer stabilization leads to accumulation of ddCas9 and appearance of aberrant parasites (Supporting Information Fig. S3B). To minimize toxicity and off‐target effects caused by over‐stabilization of ddCas9, conditional mutants were generated by incubation of parasites with 1 µM of Shld1 for 4 h.

To validate the efficiency and specificity of conditional ddCas9, we stably transfected RHddCas9 with two different sgRNA‐expression vectors (Supporting Information Table S2, Fig. S3C) targeting *gap40* (gliding‐associated protein 40) and reproduced the highly specific phenotype in the inner membrane complex (IMC) biogenesis caused by deletion of *gap40* through conventional KO strategies (Harding *et al*., 2016). In the absence of Shld1 RHddCas9‐*gap40*sgRNA clonal strains generated from the two different vectors showed no deficit. Addition of Shld1 resulted in efficient disruption of *gap40*; presenting typical *gap40KO* phenotype in ∼65% of the induced population for both clones (Supporting Information Fig. S3D). We confirmed the essentiality of *gap40* for parasite growth (Fig. 2C) and the typical collapse of the IMC (Fig. 2D), which is detected as early as 24 h after induction. Using PCR analysis we confirmed that the RHddCas9 parental strain and non‐induced RHddCas9‐*gap40* sgRNA strain had no mutations in *gap40*. In contrast, specific mutations in *gap40* gene (deletions/insertions) were identified in gDNA from RHddCas9‐*gap40*sgRNA incubated with Shld1 at the exact position targeted by the gap40 sgRNA sequence (Supporting Information Fig. S3E). As a negative control we used a sgRNA targeting an exogenous sequence not present in the *T. gondii* genome (*lacZ* gene). Neither parental RHddCas9 strain nor RHddCas9‐*lacZ*sgRNA strain showed any alteration in parasite morphology or growth in the presence or absence of Shld1 (not shown).

Disruption of *uap56* led to the same poly(A)^+^ RNA nuclear accumulation (Fig. 2E), as seen for TgUAP56 overexpression and *uap56* conditional deletion using the DiCre system (see Fig. 1). This phenotype is related to TgUAP56 function, since the absence of the protein leads to mRNA export blocking (Fig. 2F‐i, ii). This phenotype was not observed in RH‐ddCas9 or RH‐ddCas9*gap40* gRNA strains (Fig. 2E), demonstrating the specificity of gene disruption using the ddCas9 strategy. This strategy proves to be an useful tool to analyze the function of genes. However, we observed several caveats that make this tool cumbersome to use in a higher throughput scale (see Discussion).

**Figure 3:**
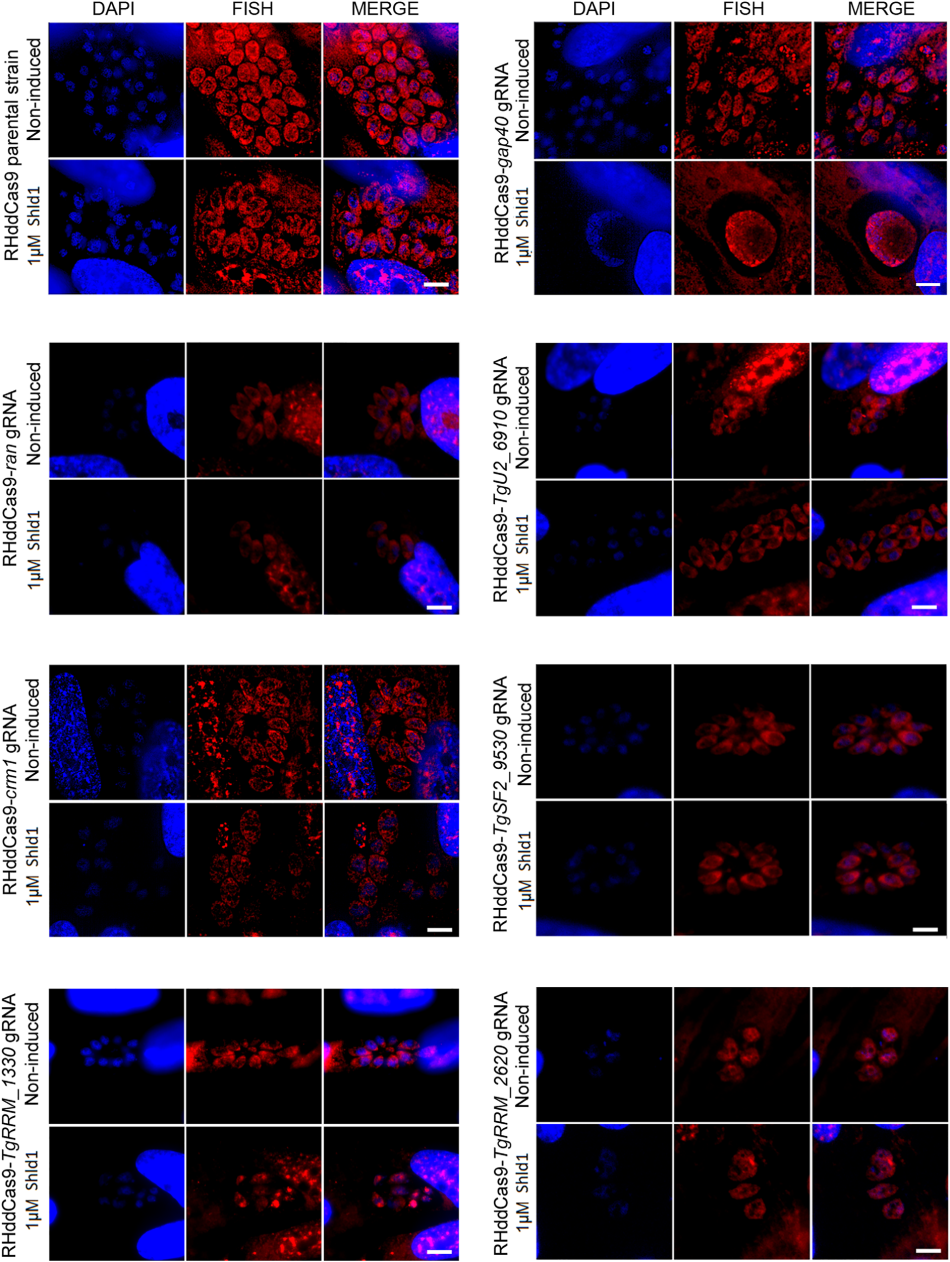
ddCas9 genetic screen for potential candidates related to mRNA export in *T. gondii*. mRNA distribution was analyzed in RHddCas9, RHddCas9‐*candidate* strains after incubation for 4 h with 1 µM Shld1 and then 48 h with fresh media. The analyses were performed by fluorescent *in situ* hybridization (FISH) using oligodT‐Alexa594 as probe, in red. Nuclear and apicoplast DNA was stained with DAPI: in blue. Scale bar: 5 µm.

Next, we stably introduced sgRNA‐expression vectors targeting each potential mRNA export candidate into RHddCas9 (Supporting Information Table S2). Interestingly only the disruption of a RNA recognition motif‐containing protein (TGME49_291330, named here as TgRRM_1330) resulted in a similar phenotype as observed for disruption of *uap56* (Fig. 3). In this case, we observed severe nuclear accumulation of poly(A)^+^ RNA ultimately leading to the death of the parasite (not shown). In RHddCas9‐*uap56* gRNA and RHddCas9‐*TgRRM_1330* gRNA strains mRNA export blocking events were up to 46% and 38% of vacuoles respectively. In contrast, components of the Ran‐dependent export pathway do not seem to be required for mRNA export in *T. gondii* (Fig. 3).

### Identification of a RNA recognition motif‐containing protein as a novel factor for mRNA export in *T. gondii*

**Figure 4:**
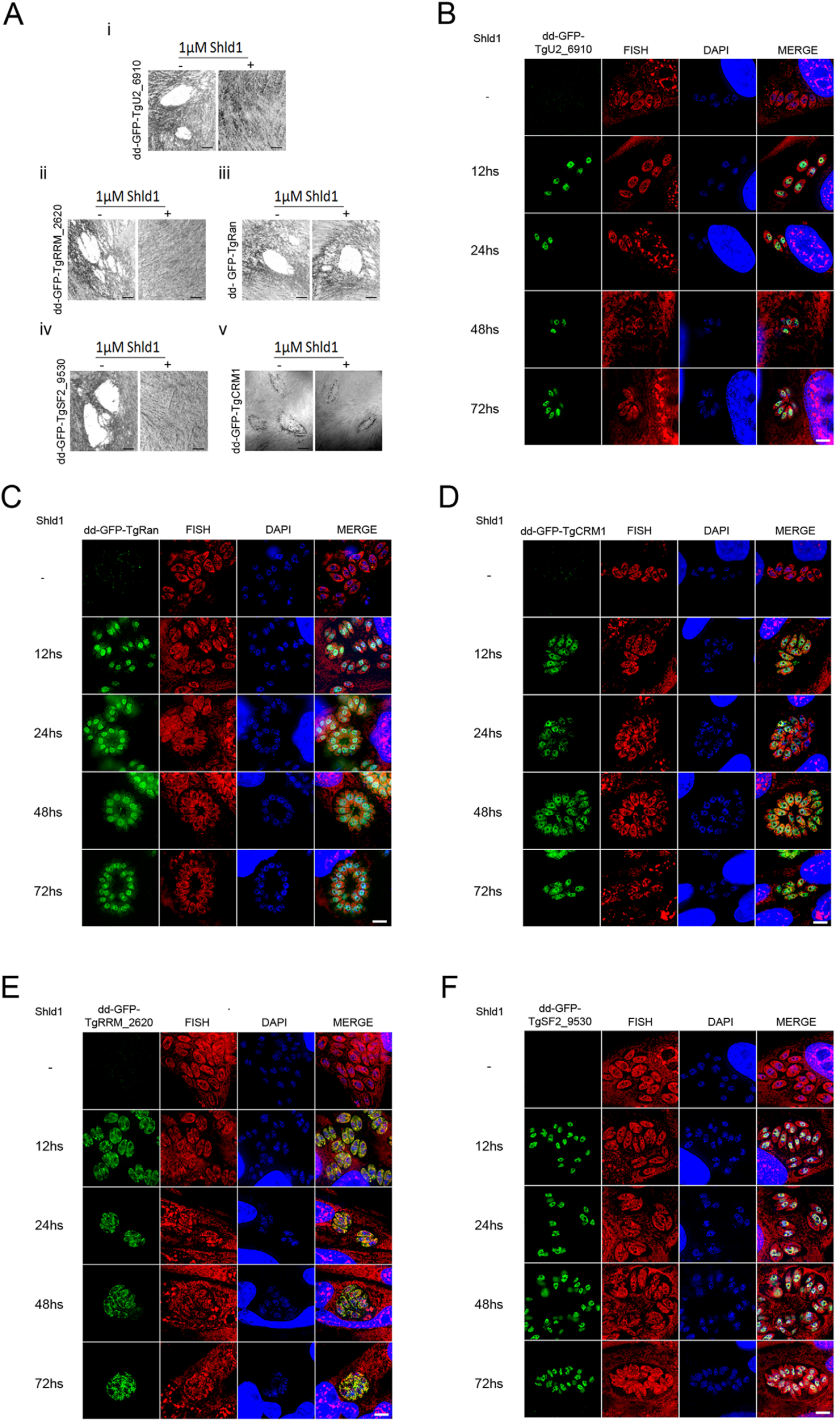
Subcellular localization and phenotypic analysis after overexpression of *T. gondii* candidate proteins in *T. gondii*. A. Plaque assays for overexpression strains. Both parasite strains were grown on human foreskin fibroblasts, in the presence or absence of 1 µM of Shield, as indicated, for 108 h. −, not induced; +, induced. Scale bar: 500 µm. B–F. mRNA distribution in different times of candidates overexpression by incubation with 1 µM of Shld1. poly(A)^+^ mRNAs were detected by fluorescent *in situ* hybridization (FISH) using oligodT‐Alexa594 as probe: in Red. Nuclear and apicoplast DNA staining with DAPI: in blue. dd‐GFP‐candidates: in green. Scale bar: 5 µm.

**Figure 5:**
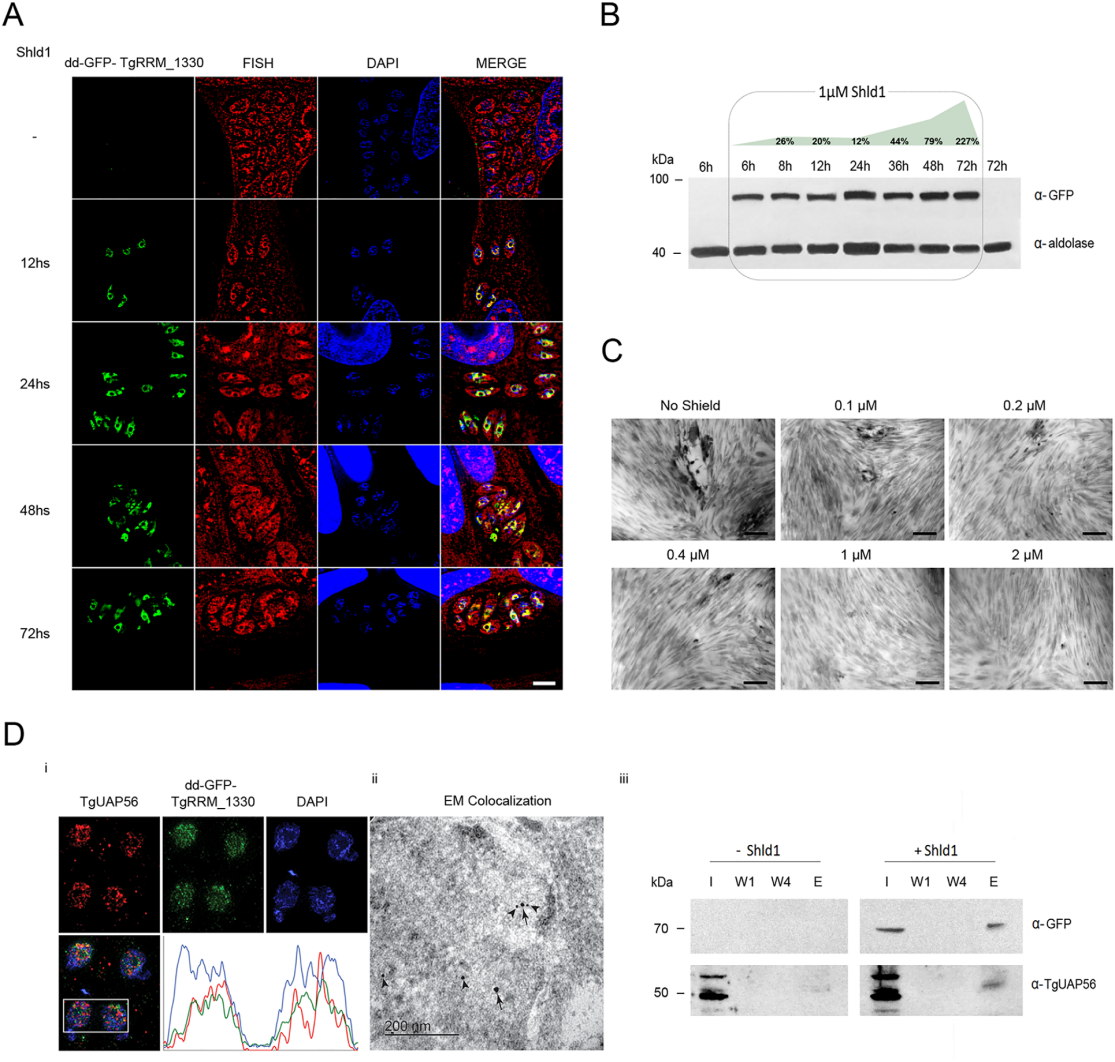
Localization and functional analysis of TgRRM_1330. A. Analysis of mRNA distribution during dd‐GFP‐ TgRRM_1330 overexpression after incubation with 1 µM Shld1 at different times. To check mRNA distribution, poly(A)^+^ mRNAs were detected by fluorescent *in situ* hybridization (FISH) using oligodT‐Alexa555 as probe: in Red. Nuclear and apicoplast DNA staining with DAPI: in blue. dd‐GFP‐ TgRRM_1330: in green. Scale bar: 5 µm. B. dd‐GFP‐TgRRM_1330 protein was detected with anti‐GFP and the overexpression levels were quantified by comparison with aldolase levels. dd‐GFP‐TgRRM_1330 was stabilized after 6 h of incubation with 1 µM of Shld1. The numbers above the Western blot means the percentage of overexpression at each indicated time point, related to result after 6 h, proportional to loading control. C. dd‐GFPTgRRM_1330 strain growth assay. Parasites were grown on human foreskin fibroblasts in the presence of different concentration of Shld1. After 108 h of incubation, the cells were fixed and stained with Giemsa. D. Colocalization analysis between TgUAP56 and dd‐GFP‐ TgRRM_1330. i) Super‐resolution microscopy of four tachyzoites nuclei. There is a clear co‐stain with anti‐TgUAP56 (in red) and the GFP signal of dd‐GFP‐TgRRM_1330 (in green) within the nucleus, stained in blue with DAPI. The fluorescence signals were analyzed and plotted, showing a co‐localization of the fluorescences in the same areas within the nuclei highlighted in the white box. ii) Immunoelectron micrograph of a tachyzoite nucleus. Arrows indicate labeling of anti‐TgUAP56 protein, and arrowheads indicate anti‐GFP protein. iii) dd‐GFP‐TgRRM_1330 immunoprecipitation. dd‐GFP‐TgRRM_1330 was stabilized for 2 h with 0,5 µM of Shld1. The membrane was incubated with anti‐TgUAP56. I: Input. W1: First wash. W4: Fourth and last wash. E: Eluted.

The results obtained by ddCas9 dependent disruption were further confirmed by overexpression studies, based on ddFKBP‐GFP as described above for TgUAP56. For most of the candidates analyzed, overexpression also resulted in lethal phenotypes without any mRNA export defects (Fig. 4). Concomitant with the results obtained for ddCas9‐mediated gene disruption, the overexpression of TgRRM_1330 resulted in mRNA export defect (Fig. 5A and B) and consequent death of parasites (Fig. 5C), as observed for TgUAP56 overexpression. Even though TgRRM_1330 sequence is not conserved, the protein contains a RNA binding domain that is conserved in orthologs of Yra1/Aly, an essential component of mRNA export in yeast (Supporting Information Fig. S4), and the experimental data suggest that TgRRM_1330 is potentially a highly divergent functional homologue of Yra1/Aly.

In summary, disruption and overexpression of CRM1 and GTPase Ran (components of Ran‐dependent pathway) did not show any mRNA export defect in *T. gondii*. However, TgUAp56 and TgRRM_1330 were found to be shown to be essential for bulk mRNA export, suggesting that *T. gondii* operate in a Ran independent pathway as found in higher eukaryotes.

### TgUAP56 and TgRRM_1330 form a functional complex

To test the hypothesis that TgRRM_1330 is a partner of TgUAP56 we performed co‐localization and immunoprecipitation analysis. For this purpose, we imaged the nucleus of tachyzoites with super‐resolution and immune‐electron microscopy. Maximum projection of SR‐SIM image of TgUAP56 (in red) and dd‐GFP‐TgRRM_1330 (in green) show a high co‐localization rate, with a similar distribution over the nucleus (stained with DAPI, in blue). The graph shows the same localization of the red signal over the green signal, both together with the localization of the DAPI signal (Fig. 5D‐i). Ultrastructural observation showed a labeling of TgUAP56 (arrows) together with TgRRM_1330 (arrowheads) near areas of dense chromatin in the nucleus of *T. gondii* tachyzoites (Fig. 5D‐ii). The interaction between dd‐GFP‐TgRRM_1330 and TgUAP56 was furthermore confirmed by immunoprecipitation (Fig. 5D‐iii). This interaction was not observed with dd‐GFP (control strain) or with dd‐GFP‐TgCRM1, as expected (data not shown).

## Discussion

mRNA export is well studied in higher eukaryotes where TREX complex has an important role, including UAP56 and adaptor proteins (for review see (Muller‐McNicoll and Neugebauer, 2013)). However, several proteins involved in this pathway are not conserved throughout the eukaryotic phylogeny with the exception of UAP56, that is also conserved in early divergent eukaryotes (Serpeloni *et al*., 2011b). We previously demonstrated that this protein is a component of mRNA export pathway in trypanosomes (Serpeloni *et al*., 2011a).

Here we confirmed the UAP56 ortholog in *T. gondii*, namely TgUAP56. The most divergent sequence is the Tryp‐Sub2 ortholog (66.1% similarity). Considering that Tryp‐Sub2 is a component of the mRNA export pathway (Serpeloni *et al*., 2011a), it would be reasonable to hypothesize that TgUAP56 has the same role as a basic component of the mRNA export pathway. However, phylogenic relationship is not a guarantee for functional conservation. In this work we present experimental data supporting UAP56 is an essential factor for mRNA export in *T. gondii*. TgUAP56 is exclusively nuclear and dispersed all over the nuclei in a punctuate pattern, similar to observations in other eukaryotes (Gatfield *et al*., 2001; Sahni *et al*., 2010; Serpeloni et al., 2011a). TgUAP56 overexpression results in a dominant negative phenotype leading to parasite death due to a block of mRNA export. Similarly, overexpression of UAP56 in *Caenorhabditis elegans* and human cells impairs mRNA export and leads to nuclear retention of poly(A)^+^ mRNAs (Luo *et al*., 2001; Strasser and Hurt, 2001; MacMorris *et al*., 2003).

The specificity of the overexpression effect was confirmed by conditional knockout for *uap56*, where nuclear accumulation of poly(A)^+^ RNAs was prominent after 48 h and consequently parasites were unable to grow and died within the host cell. These results agree with previous observations in yeast, *Drosophila melanogaster* and trypanosomes where the depletion of UAP56 orthologs resulted in growth arrest and robust accumulation of poly(A)^+^ mRNAs within the nucleus (Gatfield *et al*., 2001; Strasser and Hurt, 2001; Serpeloni et al., 2011a). Together these data demonstrate the essential role of TgUAP56 in mRNA export in *T. gondii*.

We also investigated if TgUAP56 is involved in mRNAs splicing since this protein was originally associated with the splicing machinery (reviewed in Linder and Stutz, 2001 (Linder and Stutz, 2001)). Our approach was based on the strategies used previously for the characterization of the splicing factor TgRRM1 (Suvorova *et al*., 2013), where the authors showed that a selected list of genes of *T. gondii* were mis‐spliced in the absence of TgRRM1. Importantly, we did not observe any interference in mRNA splicing of these targets in the absence of TgUAP56. Therefore, TgUAP56 appears to be exclusively required for mRNA export, potentially releasing spliced mRNAs from adaptor proteins. This idea is corroborated by studies in other model organisms that indicate that Sub2/UAP56 also plays an essential role in mRNA export (Gatfield *et al*., 2001; Jensen *et al*., 2001; Luo *et al*., 2001; Strasser and Hurt, 2001; Dias *et al*., 2010; Steckelberg and Gehring, 2014).

Since UAP56 is a necessary and specific component of a specialized mRNA export complex in mammals, the identification of homologous proteins in parasites would point to the presence of a similarly specialized pathway in apicomplexans. For this purpose, we decided to use a genetic screen based on the Cas9/CRISPR system. In *T. gondii*, CRISPR/Cas9 technology has been used successfully in several occasions (Shen *et al*., 2014; Sidik *et al*., 2014). However, constitutive expression of Cas9 in *T. gondii* seems to be toxic. In higher eukaryotes, different strategies for controlling the activity of Cas9 have been employed including the tetracycline inducible system (Zhu *et al*., 2014), splitting the Cas9 in half (Wright *et al*., 2015; Zetsche *et al*., 2015) and, very recently, a conditional Cas9 system based on the fusion with a destabilization domain (FKBP12‐L106P) (Geisinger *et al*., 2016).

Here we succeeded to generate parasites that allow regulation of Cas9 activity using the ddFKBP‐system (Herm‐Gotz *et al*., 2007) and we show that specific conditional disruption of essential genes is feasible using this approach. Our phenotypic assays using ddCas9 demonstrated that it allows the identification of specific phenotypes. As a proof of concept, we reproduced the phenotype caused by deletion of gap40 (Harding *et al*., 2016) and confirmed that disruption of this gene using ddCas9 causes the typical collapse of the IMC, while mRNA export is not affected. In contrast, disruption of *uap56* caused a block in mRNA export, corroborating our previous findings after conditional deletion of the gene using DiCre and overexpression analysis.

However, while ddCas9 can be successfully employed to screen for specific phenotypes, such as mRNA export as shown here, it should be used with caution, when screening for general growth phenotypes. In our experience disruption of non‐essential genes resulted sometimes in abnormal parasites (data not shown), a phenomenon we are currently investigating. Furthermore, overexpression of ddCas9 over extended periods results in parasites with aberrant morphology, further complicating employment of this system. Therefore, a thorough downstream analysis using additional reverse genetic tools is required to confirm observed phenotypes. Despite these disadvantages, the ddCas9 system allowed us to efficiently analyze different candidates.

The selection of candidates for genetic analysis was based on searching for orthologs of proteins that have been described in mRNA export in opisthokonts. Among all the proteins analyzed, only the ortholog of Mex67 was not identified. In this case, PSI‐BLAST searches resulted in spurious hits corresponding to only the Leucine‐rich region of Mex67, no hits corresponding to NTF2‐Like and TAP C‐terminal domains were found. It may not be surprising since Mex67 is not a highly conserved protein in divergent groups (Kramer *et al*., 2010; Serpeloni *et al*., 2011b). The only description of Mex67 in protozoa shows that it is a divergent protein with an essential motif that is absent from all other Mex67 orthologs known (Kramer *et al*., 2010; Dostalova *et al*., 2013). Consequently, all the candidates bar Mex67 were studied by functional interference. Our results showed that TgU2_6910, TgRRM_2620, and TgSF2_9530, *T. gondii* orthologs of factors involved in Ran‐independent mRNA export pathway in opisthokonts, are not crucial for mRNA export although they are essential proteins for parasite survival. These data point to the lack of a conserved mRNA export pathway in *T. gondii*. Interestingly, the lack of a conserved pathway has also been observed for the protozoa *Trypanosoma brucei* albeit Mex67 is a functional mRNA receptor in these parasites. Some authors have proposed the hypothesis of an evolutionarily divergent mechanism for mRNA export (Schwede *et al*., 2009; Dostalova *et al*., 2013; Obado *et al*., 2016) and a shared platform for transport for rRNA and mRNA has been suggested (Neumann *et al*., 2010; Buhlmann *et al*., 2015).

Based on this, we decided to address if mRNA export in *T. gondii* would be dependent on exportin (CRM1) and GTPase Ran since they are conserved proteins throughout eukaryote phylogeny and there is evidence of CRM1/Ran involvement in export of specific mRNA and rRNA (for review see (Kohler and Hurt, 2007)). The disruption of both genes did not block bulk mRNA export, suggesting that it is potentially a Ran‐exportin independent route. These results together with the identification of TgUAP56 indicate the presence of a specific mRNA export pathway as described for other organisms. We still cannot affirm that Mex67 is absent in *T. gondii* but undoubtedly the protein structure is very distinct of the receptors described so far.

Our genetic approaches identified TgRRM_1330, a RNA binding protein that is essential and a specific component of mRNA export in *T. gondii*. Interestingly, this protein contains a RNA‐binding domain that is also present in Yra1/Aly, a component of TREX that interacts with UAP56 orthologs in opisthokonts (Strasser and Hurt, 2000; Luo *et al*., 2001; Dufu *et al*., 2010). In good agreement with this, we demonstrate that TgRRM_1330 interacts with TgUAP56 in *T. gondii*. TgRRM_1330 was not detected using standard BLAST searches, however using PSI‐BLAST with 2 iterations it was possible to recover this yeast Yra1 ortholog candidate with a significant E‐value (5e‐17). The identity of TgRRM_1330 with the yeast (Yra1) and human (Aly) protein is only 21.5% and 24.9% respectively, supporting the idea that mRNA export pathway in *T. gondii* might contain divergent components.

Indeed, apicomplexans and other parasites have acquired particular features in relation to mRNA metabolism during evolution and further evidence is required to check if the presence of divergent components would correspond to distinct mechanisms of mRNA export in comparison with the pathway of opisthokonts. Our results provide the first insights into components of mRNA export in apicomplexan parasites and the description of interacting partners that are crucial for the divergent pathway. These essential proteins can serve as a handle to identify interacting proteins in further investigations.

## Experimental procedures

### Identification of candidate genes in *T. gondii*

To identify putative ortholog proteins involved in the RNA export pathway in *T. gondii*, we used previously described proteins in the literature from yeast, human and *P. falciparum* as query sequences. The list of queries used is as following: Sub2 (GI: 6320119), Yra1 (GI: 6320589), Mex67 (GI: 6325088), GTPase Ran GSp1 (GI: 6323324), and CRM1 (GI: 398366207) from yeast; U2AF35 (GI: 68800128) from human; and Npl3 (PF10_0217) and Gbp2 (PF10_0068) from *P. falciparum*. We performed BLASTP searches with E‐value threshold of 1e‐03. To be considered orthologs in *T. gondii*, identified proteins should satisfy the reciprocal best hit criteria. Alternatively, PSI‐BLAST with inclusion threshold of 0.005 was used to identify a candidate ortholog sequence of yeast Yra1 in *T. gondii*.

### Identification of orthologs genes in eukaryotes

Putative ortholog proteins identified in *T. gondii* were used as query sequences in BLASTP searches (E‐value threshold 1e‐03) against the RefSeq database to identity orthologs in 43 representative species from the major eukaryotic groups as listed on Supporting Information Table S1: Metazoans, Fungi, Amoebozoa, Plants, Apicomplexans, Kinetoplastids and Parabasalids. To be considered orthologs, identified proteins should satisfy the reciprocal best hit criteria.

### Sequence and domain analysis

Identity and similarity percentages were obtained using needle program from the EMBOSS package (Rice *et al*., 2000), which finds the optimal global alignment of two sequences. Protein domain searches were performed running hmmscan program from HMMER package (Eddy, 1998) against the collection of Hidden Markov models downloaded from the Pfam database version 27.0 (Finn *et al*., 2014).

### Phylogenetic analysis

Multiple sequence alignments of orthologs sequences were done using MAFFT version 7 with the following parameters: – localpair – maxiterate 1000 – reorder (Katoh and Standley, 2013). Phylogenetic analysis was conducted using the approximately maximum likelihood method implemented in the FastTree 2.1 program (Price *et al*., 2010) with default parameters. The tree was rendered using FigTree v1.4 (http://tree.bio.ed.ac.uk/software/figtree/).

### Cloning of DNA constructs

All oligonucleotides used in this study are listed in Supporting Information Table S2.

The TgUAP56 gene swap‐vector (*loxPUap56loxP‐mCherry‐HX*) was generated by cloning *uap56* ORF (TGME49_216860), amplified directly from cDNA with F/R primers using ApaI and loxP‐NsiI sites respectively. The amplified fragment was placed upstream of *mcherry* sequence, replacing tub8 of parental plasmid (unpublished vector). *Uap56* 5′UTR was amplified from genomic DNA (gDNA) with F/R primers using KpnI and loxP‐ApaI sites respectively. The amplified fragment was placed upstream of the *uap56* cDNA for the transcription of *uap56* gene to be driven by the endogenous promoter present in 5´UTR. Finally, 3′UTR of *uap56* was amplified from gDNA using F/R primers using SacI restriction site for cloning. The plasmid was linearized with KpnI before RH DiCre ΔKu80 strain transfection.

To generate conditional ddCas9 plasmid (*p5RT70DDmycFlagCas9*) the synthetic *cas9* expressing cassette from Lourido's lab (Addgene ID 52694 (Sidik *et al*., 2014)) was cloned into plasmid *p5RT70ddmycGFPPfMyoAtailTy‐HX* (Hettmann *et al*., 2000) using NcoI‐NotI restriction sites. The plasmid was linearized with NotI before transfection into the RH Δ*hxgprt* strain.

A single guide RNA (sgRNA) cassette for each candidate synthesized by GeneScript was inserted into a plasmid including the DHFR resistant cassette (*pU6‐gRNA‐crisprRNA*). The 20 nucleotides of the guide RNA (gRNA) were designed using the online software E‐CRISP (http://www.e-crisp.org/E-CRISP/designcrispr.html) aiming for exons close to the 5′ end and maintaining NGG as PAM sequence. The plasmids were linearized with NotI before transfection into the ddCas9 strain.

The Overexpression vectors were generated by cloning each ORF (TgUAP56 (TGME49_216860), TgCRM1 (TGME49_249530), TgSF2_9530 (TGME49_119530), TgRRM_2620 (TGME49_062620), TgRan (TGME49_248340), TgRRM_1330 (TGME49_291330), TgU2_6910 (TGME49_236910)), amplified directly from cDNA with F/R primers using appropriate restriction sites, into *p5RT70ddmycGFPPfMyoAtailTy‐HX* (Hettmann *et al*., 2000). The plasmids were linearized with KpnI before transfection into the RH Δ*hxgprt* strain.

### Parasite parental and transgenic strains

*Toxoplasma gondii* tachyzoites (RH Δ*hxgprt –* RH strain (Donald *et al*., 1996) and RH DiCre ΔKu80 strain ‐ DiCre strain (Pieperhoff *et al*., 2015), and transgenic strains generated in this study) were cultured on human foreskin fibroblasts (HFF) and maintained in Dulbecco's modified Eagle's medium (DMEM) supplemented with 10% fetal calf serum, 2 mM glutamine and 25 μg/mL gentamicin.

Freshly released parasites (∼5 × 10^7^) were transfected and selected in presence of mycophenolic acid and xanthine (Donald *et al*., 1996) or pyrimethamine to generate stable lines as previously described (Donald and Roos, 1993).

RH Δ*hxgprt* parasites were transfected individually with overexpression plasmids to generate clonal lines for overexpression of all candidates.

The RH DiCre ΔKu80 strain was transfected with *loxPUap56loxP‐mCherry‐HX* plasmid to generate TgUAP56 knockout strain (cKOuap56) based on a previously used gene‐swap strategy (Andenmatten *et al*., 2013).

RHΔ*hxgprt* parasites were transfected with *p5RT70DDmycFlagCas9* plasmid to generate a conditional Cas9 expressing strain (ddcas9). This parental cell line was transfected with individual synthetic *pU6‐gRNA‐crisprRNAs* plasmids (listed on Supporting Information Table S2) to generate ddCas9 strains for each target candidate.

### Analysis of mutations caused by ddCas9/CRISPR targeting

Genomic DNA was extracted from induced and non‐induced parasites using the DNeasy Blood and Tissue kit following manufacturer procedures (Qiagen cat# 69506). PCR primers flanking the predicted target site were used to amplify amplicons using High Fidelity Platinum® Taq DNA Polymerase (ThermoFisher cat# 11304‐011), PCR primers listed in Supporting Information Table S2. Amplicons were cloned into pGEM‐T Easy Vector System (Promega cat# A1360) using standard cloning and amplification procedures prior to sequencing (LIGHTRUN^TM^ Sequencing Service at GATC, Germany) using T7 and SP6 primers (Supporting Information Table S2). Sequences were analyzed using BioEdit software version 7.2.5. (Tom Hall. Ibis Biosciences. Carlsbad, CA, USA).

### Immunoblot assays

Parasites were incubated in culture media supplemented with or without 1 μM Shld1 (overexpression assays and Cas9‐mediated disruption assays) or 50 nM of rapamycin (inducible knockout assay). Protein extracted from parasites were prepared for Western blot analysis as described previously (Hettmann *et al*., 2000), using 12% polyacrylamide gels under reducing condition with 100 mM DTT. Equal number of parasites was loaded per experiment. Polyclonal anti‐Tryp‐Sub2 (1:1000) (Serpeloni *et al*., 2011a), monoclonal anti‐GFP (1:500) (Roche, #cat11814460001) and monoclonal anti‐flag (1:500) (Fisher/Thermo Scientific, #cat 11525702) antibodies were used for specific protein detection respectively. Monoclonal anti‐aldolase (1:10.000) (Starnes *et al*., 2006) was used as loading control. ImageJ software with the densitometry plugin (Version 1.6, National Institutes of Health, Bethesda, MD) was used for protein quantification.

### Fluorescent *in situ* hybridization (FISH) and immunofluorescence assays

Intracellular parasites were grown in the absence or presence of 0.1‐2 μM Shld1 (overexpression assays), 1 μM Shld1 (Cas9‐mediated disruption assays) or 50 nM of rapamycin (inducible knockout assay). To detect poly(A)^+^ RNA in *T. gondii* FISH assays were performed as previously described (Lirussi and Matrajt, 2011, Serpeloni *et al*., 2011a). Tachyzoite‐infected HFF cells grown on glass coverslips were fixed with 4% formaldehyde in PBS for 20 min at room temperature and permeabilized with 0.2% Triton X‐100 in 2X SSPE (SSPE 2X: 300 mM NaCl, 20 mM phosphate buffer pH 7.4, 2 mM EDTA) for 20 min. The parasites were washed three times with 2X SSPE and blocked with hybridization solution (HS) (10% Dextran, SSPE 2X, 35% formamide, 0.5 mg/mL tRNA) for 30 min at 37°C in a humidified chamber. Further, 1ng/µL of the probe (oligodT conjugated with Alexa488 or Alexa555, synthesized by Invitrogen) was added in HS and denatured for 3 min at 65°C before hybridization with the cells. The cells were incubated with the probe for 16 h at 37°C in the humidified chamber. After the hybridization, the cells were washed with 2X SSC followed by 1x SSC for 15 min each (1x SSC: 0.15M NaCl, 0.015M Na_3_Citratex‐2H_2_O pH 7.4).

For IF assays, cells were blocked and permeabilized with PBS containing 3% bovine serum albumin and 0.2% Triton X‐100 for 20 min before incubation with primary antibody for 1 h at RT. Anti‐TrypSub2, (Serpeloni *et al*., 2011a), anti‐IMC1 or IMC3 (1:1000) and anti‐flag (1:200) (Fisher/Thermo Scientific, #cat 11525702) antibodies were used for immunolocalization. Post primary antibody hybridization Cells were incubated with the secondary antibodies (Alexa 594‐conjugated anti‐rabbit (Invitrogen, #cat A11012); Alexa 488 or 594‐conjugated goat anti‐mouse antibodies (Invitrogen #cat A‐11001 and A11005, respectively)) diluted to 1:3000 and incubated for 1 h at RT. Cells were washed (buffer) several times and mounted onto slides with DAPI Fluoromount (Southern Biotech UK, #cat 0100‐20). We combine immunofluorescence and FISH assays. For this, the immunofluorescence protocol was used and a fixation step with PFA 4% fixation followed by incubation oligodT conjugated with Alexa (as described for FISH) was included before the incubation with secondary antibodies.

Image acquisition was conducted using a 100× or 63× oil objective on a Zeiss Axioskop 2 fluorescence microscope with an Axiocam MRm CCD camera using Zen software (Zeiss). Further image processing was performed using ImageJ 1.34r software and Photoshop CS6 (Adobe Systems Inc). Immunofluorescence signals quantification was performed with ImageJ software with densitometry plugin (Version 1.6, National Institutes of Health, Bethesda, MD). Super‐resolution structure illumination microscopy was performed using a Zeiss Elyra PS.1 super‐resolution microscope equipped with a sCMOS PCO camera. The Plan‐Apochromat 63×/1.4 Oil DIC lens was used, and Z‐stacks were acquired in five rotations using the ZEN Black Edition Imaging software (Zeiss). Images were then processed in ZEN Black Edition Imaging software (Zeiss), using the structural illumination manual processing tool, with a noise filter of −6.0, and an output as SR‐SIM. Colocalization and fluorescence intensity analysis were carried out in FIJI software (Schindelin *et al*., 2012).

### Immunoelectron microscopy

For the immunocytochemistry, infected cells were fixed overnight in 4% paraformaldehyde and 0.2% glutaraldehyde in phosphate buffer. The samples were washed in phosphate buffer and dehydrated in ascending ethanol solutions. After a progressive infiltration process with LR White resin, the polymerization was carried out in gelatin capsules under ultraviolet light. Formvar‐coated nickel grids with ultrathin sections were incubated with blocking solution (3% BSA, 0.02% Tween 20, in phosphate buffer) for 1 h. The grids were incubated with the primary antibody anti‐GFP (1:20, Roche, #cat11814460001) diluted in blocking buffer for 1 h followed by several washes in blocking buffer. The grids were then incubated with 10 nm gold‐conjugated anti‐mouse secondary antibody (1:10, Aurion, the Netherlands) for 1 h, followed by several washes with blocking buffer. The grids were incubated with anti‐TrypSub2 primary antibody (1:50, polyclonal (Serpeloni *et al*., 2011a)) for 1 h, followed by several washes in blocking buffer. The grids were then incubated with 15 nm gold‐conjugated Protein A (Aurion, the Netherlands) for 1 h, followed by washes in blocking buffer and phosphate buffer. The material was stained with uranyl acetate prior observation in a Tecnai T20 transmission electron microscope (FEI, the Netherlands). Images were analyzed and processed in FIJI (Schindelin *et al*., 2012) and Adobe Photoshop.

### Real‐time PCR (qPCR)

Total RNA was isolated in duplicate from RH DiCre ΔKu80 and cKOuap56 strains incubated with 50 nM rapamycin at different points using RNeasy® Mini Kit (Qiagen) according to the manufacturer's direction and MIQE criteria (Bustin *et al*., 2009). Contaminating DNA was digested with 1 U DNAse RNAse‐free (Promega) per µg RNA. 1 µg RNA was reverse transcribed using random primers (Invitrogen) and ImProm‐II™ Reverse Transcription System (Promega) as default protocol. To access *uap56* mRNA levels, we performed real‐time PCR reactions in triplicate using SYBR green master mix (Applied Biosystems) on AB7500 (Applied Biosystems, Invitrogen). mRNA levels were normalized to reference tubulin mRNA levels, using primers described previously (Dalmasso *et al*., 2009). The relative expression levels were calculated based on the Livak method (Livak and Schmittgen, 2001) and percentage of *uap56* mRNA levels was analyzed by media of total mRNA per time.

### Growth assays

Plaque assays were performed as described previously (Roos *et al*., 1994). Monolayers of HFF grown in 6‐well plates were infected with 200 tachyzoites per well. Parasites were incubated with 0.1–2 µM of Shld1 (overexpression assays) or 50 nM of rapamycin (inducible knockout assay). For ddCas9 assays, cells were incubated with 1 µM of Shld1, after 24 h this was replaced with fresh DMEM without Shld1. For inducible knockout assays, the cells were incubated with 50 nM of rapamycin for 24 h. After this time, the cells were washed and maintained with DMEM. After 108 h of incubation at 37°C, 5% CO_2_, cells were fixed for 10 min with 100% methanol at −20°C, stained with Giemsa for 10 min and washed once with PBS. Images were taken using a Zeiss microscope (Axiovert 200M) with a 4× objective and plaque size was determined using Axiovision software (Zeiss).

### Analysis of mRNA processing by splicing

The analysis of mRNA splicing was performed by analytical PCR for seven selected genes as previously described (Suvorova *et al*., 2013). Total RNA was purified using RNeasy® Mini Kit (Qiagen) from RH DiCre ΔKu80 and cKOuap56 strains after 24 and 48 h of incubation with rapamycin. The RNA was reverse transcribed using random primers (Invitrogen #cat 48190‐011) and the target sequences were amplified by PCR using specific primers that span an intron, as listed in Supporting Information Table S2. gDNA was used as reference to distinguish between properly spliced (S) and pre‐spliced (PS) species. mRNAs from the following genes were analyzed: RNA polymerase II *p8.2* subunit (TGME49_217560), RNA polymerase II *p19* subunit (TGME49_271300), RNA polymerase II *p23* subunit (TGME49_240590), *imc1* (TGME49_231640), *imc15* (TGME49_275670), *imc5* (TGME49_224530), and transcription factor *iid* (TGME49_258680). Tubulin was used as loading control and the primers were described previously (Dalmasso *et al*., 2009).

### Immunoprecipitation

We performed immunoprecipitation experiments using the dd‐GFPTgRRM_1330 strain to determine whether TgUAP56 co‐immunoprecipitates with dd‐GFP‐TgRRM_1330. Intracellular tachyzoites were incubated with 0.5 µM of Shld1 for 2 h and then homogenized in a salt buffer [10 mM Trisodium Citrate, 20 mM HEPES, pH 7.4, 1 mM MgCl_2_, 10 µM CaCl_2_, 0,1% CHAPS, 0,5% Nonidet P‐40 (NP‐40), phosphate inhibitor cocktail (P‐5726; Sigma, St. Louis, MO)]. The samples were centrifuged at 15,000 ×*g* for 10 min and the supernatant was incubated with α‐GFP antibody (Roche) with protein G‐agarose beads (CAT) for 2 h at 4°C. The beads were washed four times in the same buffer solution and antigens were eluted from the beads with 60 μl of 2× Laemmili SDS buffer. Samples were boiled for 5 min before SDS‐PAGE separation.

## Author contributions

Experimental design: MS, EJR, MM, and ARA; Generation of transgenic strains: MS, EJR, CK, and NA; Conditional Cas9 system establishment: EJR and MS; Image acquisition, analysis and interpretation: MS, EJR, and LL; Sequence and phylogenetic analysis: NMV; Splicing analysis: MS and PM; Manuscript writing: MS, EJR, NMV, GP, MM, and ARA.

## Supporting information

Supporting Information
