# Supplementary material for: UAP56 is a conserved crucial component of a divergent mRNA export pathway in Toxoplasma gondii
Source: Mol Microbiol. 2016 Sep 14;102(4):672–89. doi: 10.1111/mmi.13485 (PMC5118106; doi:10.1111/mmi.13485)
Supplement: Supplementary file 1 — Supporting Information [file MMI-102-672-s001.pdf]

A

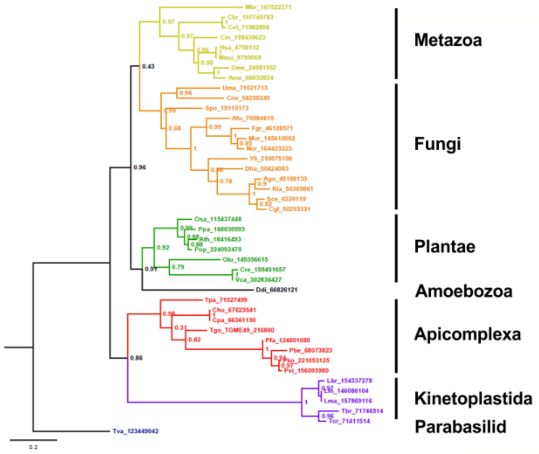

B

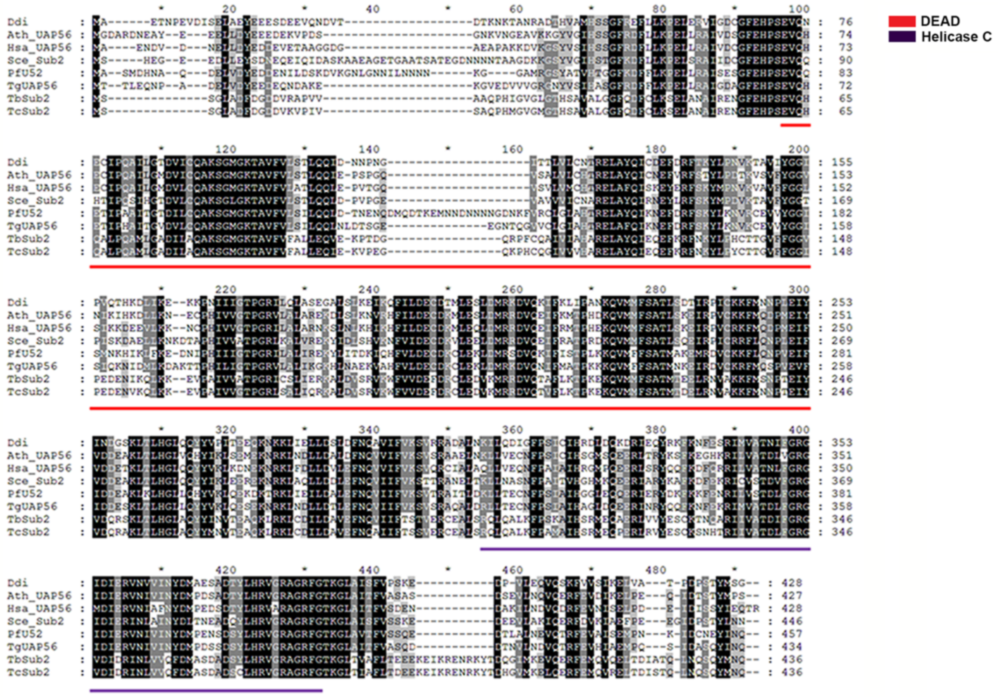

C

	Ddi	Ath_UAP56	Hsa_UAP56	Sce_Sub2	PfU52	TgUAP56	TbSub2	TcSub2
Ddi	x	78.0	77.4	71.8	70.5	74.0	65.1	65.5
Ath_UAP56	63.4	x	83.3	76.5	72.8	77.6	64.1	64.9
Hsa_UAP56	58.1	70.1	x	77.1	71.0	78.6	66.4	66.4
Sce_Sub2	54.4	61.4	61.5	x	70.9	72.8	62.7	61.5
PfU52	53.9	58.5	56.9	54.6	x	81.4	63.1	62.2
TgUAP56	57.0	65.1	64.6	56.1	68.6	x	66.1	66.1
TbSub2	44.5	48.1	48.5	43.7	44.2	47.3	x	96.1
TcSub2	45.0	49.4	49.0	44.2	44.7	47.1	89.4	x

% Similarity
% Identity

Supp. Figure 1. UAP56 is a highly conserved RNA helicase in eukaryotes. A. Phylogenetic reconstruction of UAP56 orthologous sequences across different groups of eukaryotes. Numbers at nodes are the bootstrap values. Amoebozoa (black): Ddi – *Dictyostelium discoideum*; Plants (green): Osa – *Oryza sativa*, Ppa – *Physcomitrella patens*, Ath – *Arabidopsis thaliana*, Pop – *Populus trichocarpa*, Olu – *Ostreococcus lucimarinus*, Cre – *Chlamydomonas reinhardtii*, Vca – *Volvox carteri*; Fungi (orange): Uma – *Ustilago maydis*, Cne – *Cryptococcus neoformans*, Spo – *Schizosaccharomyces pombe*, Afu – *Aspergillus fumigatus*, Fgr – *Fusarium graminearum*, Mor – *Magnaporthe oryzae*, Ncr – *Neurospora crassa*, Yli – *Yarrowia lipolytica*, Dha – *Debaryomyces hansenii*, Ago – *Ashbya gossypii*, Kla – *Kluyveromyces lactis*, Cgl – *Candida glabrata*, Sce – *Saccharomyces cerevisiae*; Metazoans (yellow): Cbr – *Caenorhabditis briggsae*, Cel – *Caenorhabditis elegans*, Cin – *Ciona intestinalis*, Hsa – *Homo sapiens*, Mmu – *Mus musculus*, Dme – *Drosophila melanogaster*, Ame – *Apis mellifera*, Mbr – *Monosiga brevicolis*; Parabasilid (blue): Tva – *Trichomonas vaginalis*; Kinetoplastids (purple): Lbr – *Leishmania braziliensis*, Lin – *Leishmania infantum*, Lma – *Leishmania major*, Tbr – *Trypanosoma brucei*, Tcr – *Trypanosoma cruzi;* Apicomplexans (red): Tpa – *Theileria parva*, Cho – *Cryptosporidium hominis*, Cpa – *Cryptosporidium parvum*, Pfa – *Plasmodium falciparum*, Pbe – *Plasmodium berghei*, Pkn – *Plasmodium knowlesi*, Pvi – *Plasmodium vivax*, Tgo – *Toxoplasma gondii*. **B. Multiple sequence alignment of UAP56 ortholog proteins.** This analysis shows a high conservation along the entire length of representative sequences of eukaryotes, including apicomplexans and kinetoplastids. N-terminal region containing DEAD (PF00270) domain. C-terminal region containing Helicase_C (PF00271) domain. Both domains are evidenced. The Genbank Identifier (GI) number for the entries are listed as follows: (Ddi 66826121), (Ath_UAP56 18416493), (Hsa_UAP56 4758112), (Sce_Sub2 6320119), (PfU52 124801080), (TgUAP56 237843393), (TbSub2 71746514), (TcSub2 71411514). **C. Percentage of similar (upper diagonal) and identical (lower diagonal) amino acids based on optimal global alignment between pairs of sequences showed in B**.

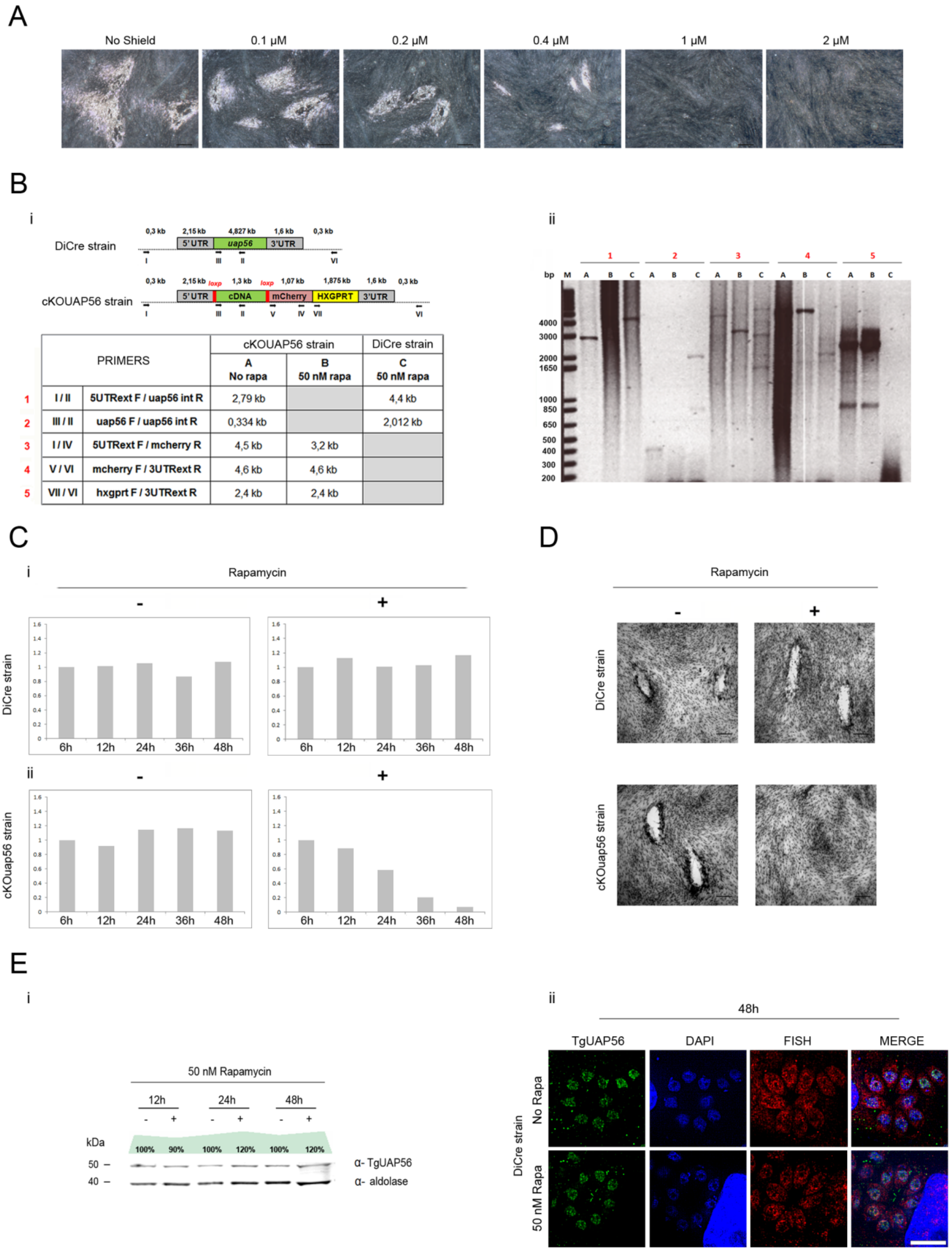

	PRIMERS		cKOUAP56 strain A No rapa	cKOUAP56 strain B 50 nM rapa	DiCre strain C 50 nM rapa
1	I / II	5UTRext F / uap56 int R	2,79 kb		4,4 kb
2	III / II	uap56 F / uap56 int R	0,334 kb		2,012 kb
3	I / IV	5UTRext F / mcherry R	4,5 kb	3,2 kb	
4	V / VI	mcherry F / 3UTRext R	4,6 kb	4,6 kb	
5	VII / VI	hxgprt F / 3UTRext R	2,4 kb	2,4 kb	

Supp. Figure 2. A. Growth assay of ddGFPTgUAP56 strain. Parasites were grown on human foreskin fibroblasts in the presence of different concentration of Shld1. After 108 hours of incubation, the cells were fixed and stained with Giemsa. **B. Genomic PCR analysis of cKOuap56 strain**. **i)** Schematic of *uap56* locus in parental DiCre strain, cKOuap56 strain and region for annealing primers. Table below: Primers and expected sizes of PCR products. Primers sequences are available in Table S2. Letters indicate parasites analyzed: A, B: cKOuap56 strain. C: DiCre strain. 50 nM of rapamycin was added for 24 hours, as indicated, to induce *uap56* knockout. Expected sizes are shown for each primer pair in kb. Gray cells indicate that no amplification is expected. Size of each region is indicated above. cDNA: *uap56* coding region. **ii)** Analytical PCR results on 1% Agarose gel. Numbers and letters are related with (i). Confirmation of clonal cKOuap56 strain by analysing the correct recombination of *uap56-LoxP* cassette in original *uap56* locus by using genomic DNA (primers set 1 and 2, A and C comparison). The integration was also confirmed by the presence of *mcherry* and *hxgprt* (primers set 3, 4 and 5). *uap56* cDNA excision was confirmed 24 hours after induction with rapamycin (primers set 1 and 2, A and B comparison). M. 1 kb Plus DNA Marker (Invitrogen). **C. Real-time RT-PCR analysis of uap56 mRNA expression in DiCre (i) and cKOuap56 (ii) strains incubated with 50 nM of rapamycin at different times.** Relative expression levels of uap56 mRNA were normalized to endogenous control (tubulin) and the values gained were *normalised* against 6 hour samples values. Bar graph representing the fold changes of mRNA levels quantified by normalization to tubulin, an internal control (n=2). **D. Plaque assays for DiCre and cKOuap56 strains.** Both parasite strains were grown on human foreskin fibroblasts, in the presence of 50 nM of rapamycin, as indicated, for 108 hours. -, not induced; +, induced. Scale bar: 500 μm**. E. TgUAP56 protein levels and bulk mRNA distribution in parental DiCre strain under rapamycin induction**. **i) TgUAP56 protein levels were analyzed in western blot after incubation with 50 nM of rapamycin at different times. -, not induced; +, induced**. Aldolase: Loading

control.The numbers above the Western blot means the percentage of TgUAP56 protein at each indicated time point, proportional to loading control. **ii) bulk mRNA distribution was analyzed in parental DiCre strain after incubation with rapamycin at 48 hours by fluorescent *in situ* hybridization (FISH) using oligodT-Alexa594 as probe, in red**. Nuclear and apicoplast DNA was staining with DAPI: in blue. Scale bar: 5 µm.

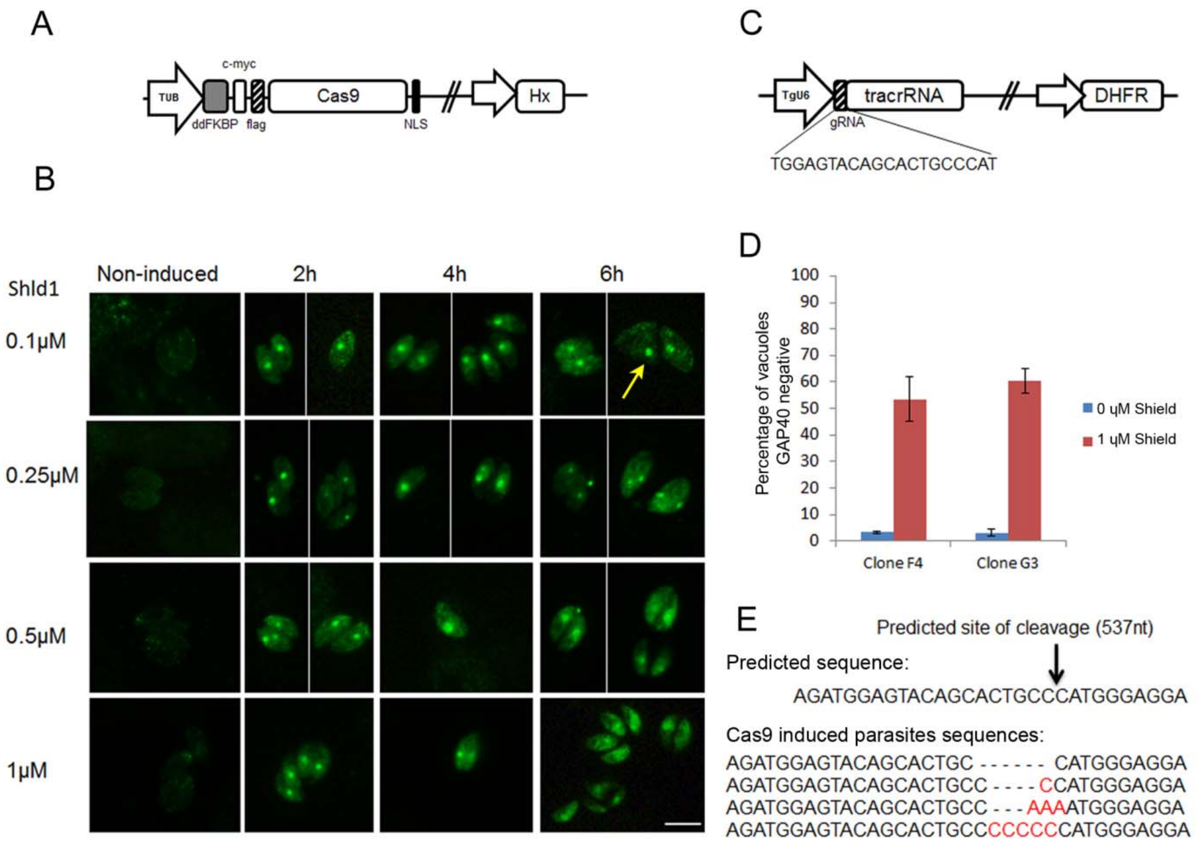

Supp. Figure 3. A. Scheme of the vector use for transfection into RHΔ*hxgprt*. Cas9 was fused to the FKBP degradation domain (dd). **B. Cas9 expression kinetics.** Expression of Cas9 starts soon after induction with Shld1 and it is localized in the nucleus having a higher concentration in nucleoli at early times of induction. Yellow arrow shows aberrant parasites Scale bar: 10 µm.**C. Schematic representation of the sgRNA carrier vector and *gap40* specific gRNA sequence. D. Efficiency of GAP40 depletion after 24 hours induction with Shld1 in two different clones. E. Predicted site of cleavage and indels (in red letters) obtained after induction with Shld1 and sequencing.**

TgU2_6910

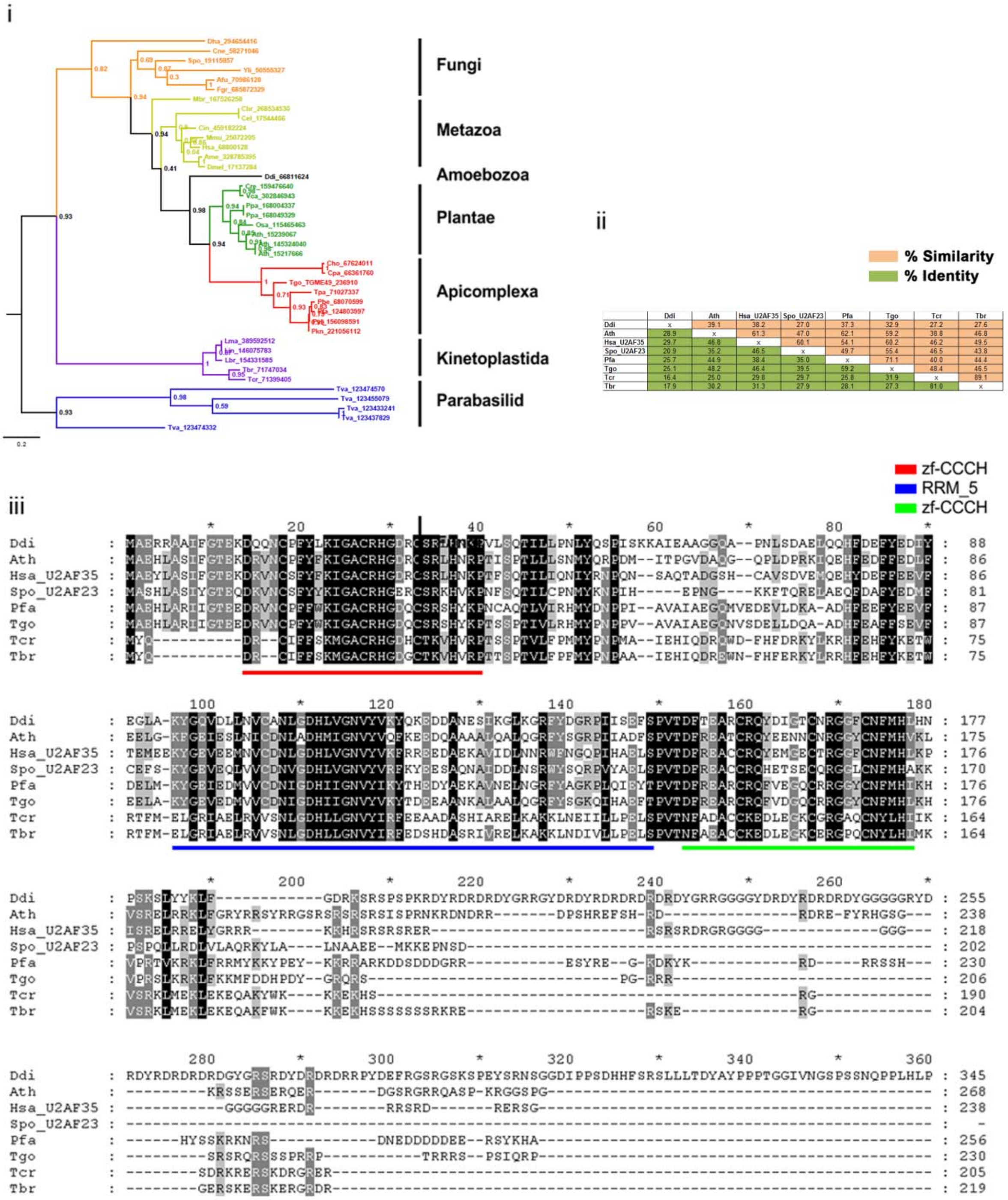

	Ddi	Ath	Hsa_U2AF35	Spo_U2AF23	Pfa	Tgo	Tcr	Tbr
Ddi	x	39.1	38.2	27.0	37.3	32.9	27.2	27.6
Ath	28.9	x	61.3	47.0	62.1	59.2	38.8	46.8
Hsa_U2AF35	29.7	46.8	x	60.1	54.1	60.2	46.2	49.5
Spo_U2AF23	20.9	35.2	46.5	x	49.7	55.4	46.5	43.8
Pfa	25.7	44.9	38.4	35.0	x	71.1	40.0	44.4
Tgo	25.1	48.2	46.4	39.5	59.2	x	48.4	46.5
Tcr	16.4	25.0	29.8	29.7	25.8	31.9	x	89.1
Tbr	17.9	30.2	31.3	27.9	28.1	27.3	81.0	x

Supp. Figure 4. TgU2_6910 is less conserved in eukaryotes. A-C. i) Phylogenetic reconstruction of orthologous sequences across different groups of eukaryotes. Numbers at nodes are the bootstrap values. Amoebozoa (black): Ddi – *Dictyostelium discoideum*; Plants (green): Osa – *Oryza sativa*, Ppa – *Physcomitrella patens*, Ath – *Arabidopsis thaliana*, Pop – *Populus trichocarpa*, Olu – *Ostreococcus lucimarinus*, Cre – *Chlamydomonas reinhardtii*, Vca – *Volvox carteri*; Fungi (orange): Uma – *Ustilago maydis*, Cne – *Cryptococcus neoformans*, Spo – *Schizosaccharomyces pombe*, Afu – *Aspergillus fumigatus*, Fgr – *Fusarium graminearum*, Mor – *Magnaporthe oryzae*, Ncr – *Neurospora crassa*, Yli – *Yarrowia lipolytica*, Dha – *Debaryomyces hansenii*, Ago – *Ashbya gossypii*, Kla – *Kluyveromyces lactis*, Cgl – *Candida glabrata*, Sce – *Saccharomyces cerevisiae*; Metazoans (yellow): Cbr – *Caenorhabditis briggsae*, Cel – *Caenorhabditis elegans*, Cin – *Ciona intestinalis*, Hsa – *Homo sapiens*, Mmu – *Mus musculus*, Dme – *Drosophila melanogaster*, Ame – *Apis mellifera*, Mbr – *Monosiga brevicolis*; Parabasilid (blue): Tva – *Trichomonas vaginalis*; Kinetoplastids (purple): Lbr – *Leishmania braziliensis*, Lin – *Leishmania infantum*, Lma – *Leishmania major*, Tbr – *Trypanosoma brucei*, Tcr – *Trypanosoma cruzi;* Apicomplexans (red): Tpa – *Theileria parva*, Cho – *Cryptosporidium hominis*, Cpa – *Cryptosporidium parvum*, Pfa – *Plasmodium falciparum*, Pbe – *Plasmodium berghei*, Pkn – *Plasmodium knowlesi*, Pvi – *Plasmodium vivax*, Tgo – *Toxoplasma gondii*. **ii) Percentage of similar (upper diagonal) and identical (lower diagonal) amino acids based on optimal global alignment between pairs of sequences showed in iii**. **iii) Multiple sequence alignment of ortholog proteins.** This analyzes show a low conservation along the entire length of representative sequences of eukaryotes, including apicomplexans. Domains are evidenced.

TgRRM_2620

i

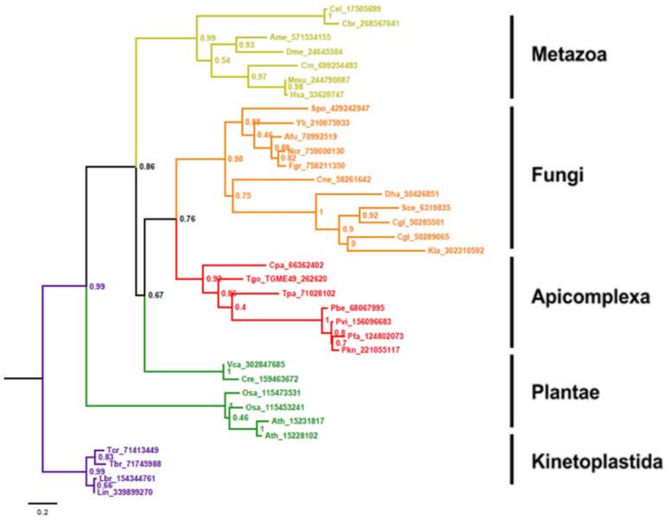

ii

% Similarity
% Identity

	Ddi	Ath	Hsa	Sce_Gbp2	Pfa	Tgo	Tcr	Tbr
Ddi	x	NC	NC	NC	NC	NC	NC	NC
Ath	NC	x	27.1	21.4	30.7	34.0	32.1	32.2
Hsa	NC	14.6	x	25.8	17.7	26.7	18.4	18.6
Sce_Gbp2	NC	13.4	15.4	x	24.4	20.4	21.6	19.3
Pfa	NC	20.5	10.5	16.8	x	45.4	37.6	39.7
Tgo	NC	25.9	19.9	13.4	33.9	x	38.3	40.8
Tcr	NC	20.9	11.5	14.4	26.1	23.0	x	77.0
Tbr	NC	19.7	11.4	12.1	23.3	25.7	66.3	x

RRM_1

iii

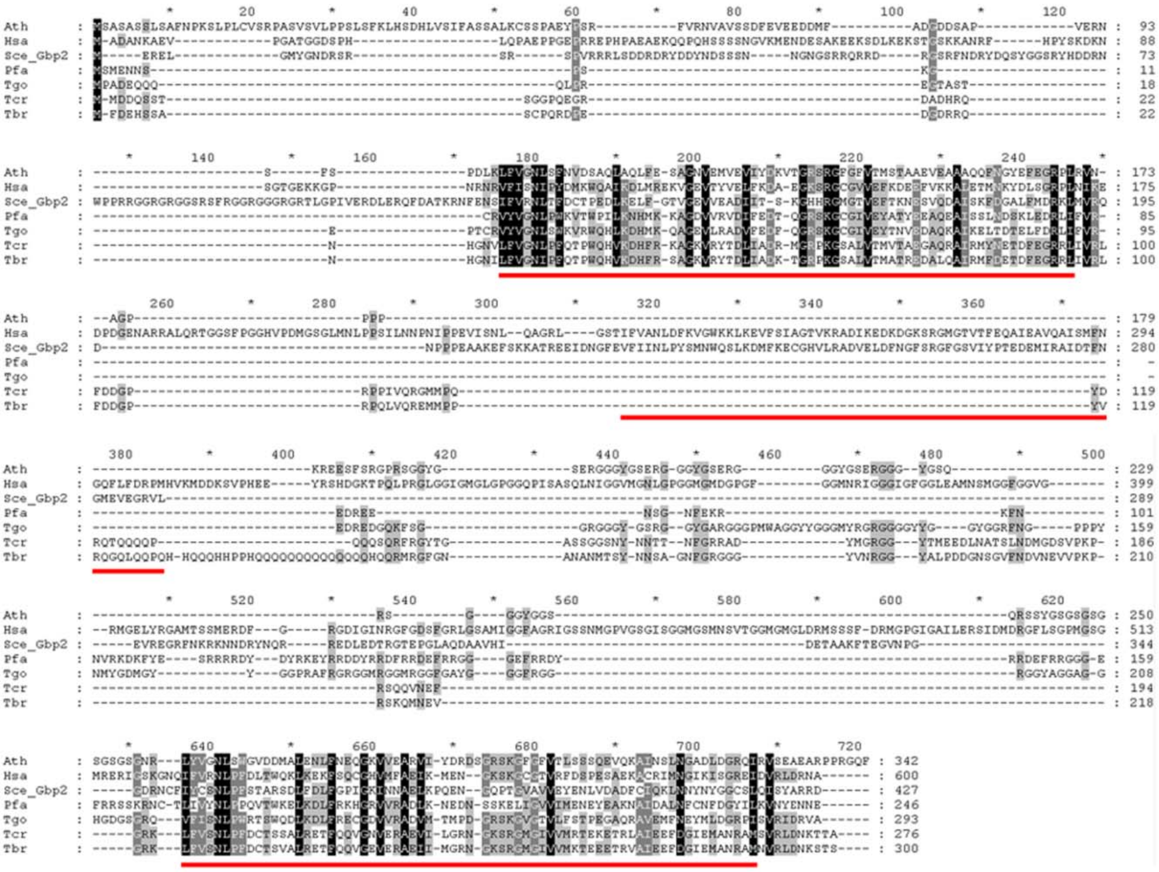

Supp. Figure 5. TgRRM_2620 is less conserved in eukaryotes. A-C. i) Phylogenetic reconstruction of orthologous sequences across different groups of eukaryotes. Numbers at nodes are the bootstrap values. Amoebozoa (black): Ddi – *Dictyostelium discoideum*; Plants (green): Osa – *Oryza sativa*, Ppa – *Physcomitrella patens*, Ath – *Arabidopsis thaliana*, Pop – *Populus trichocarpa*, Olu – *Ostreococcus lucimarinus*, Cre – *Chlamydomonas reinhardtii*, Vca – *Volvox carteri*; Fungi (orange): Uma – *Ustilago maydis*, Cne – *Cryptococcus neoformans*, Spo – *Schizosaccharomyces pombe*, Afu – *Aspergillus fumigatus*, Fgr – *Fusarium graminearum*, Mor – *Magnaporthe oryzae*, Ncr – *Neurospora crassa*, Yli – *Yarrowia lipolytica*, Dha – *Debaryomyces hansenii*, Ago – *Ashbya gossypii*, Kla – *Kluyveromyces lactis*, Cgl – *Candida glabrata*, Sce – *Saccharomyces cerevisiae*; Metazoans (yellow): Cbr – *Caenorhabditis briggsae*, Cel – *Caenorhabditis elegans*, Cin – *Ciona intestinalis*, Hsa – *Homo sapiens*, Mmu – *Mus musculus*, Dme – *Drosophila melanogaster*, Ame – *Apis mellifera*, Mbr – *Monosiga brevicolis*; Parabasilid (blue): Tva – *Trichomonas vaginalis*; Kinetoplastids (purple): Lbr – *Leishmania braziliensis*, Lin – *Leishmania infantum*, Lma – *Leishmania major*, Tbr – *Trypanosoma brucei*, Tcr – *Trypanosoma cruzi;* Apicomplexans (red): Tpa – *Theileria parva*, Cho – *Cryptosporidium hominis*, Cpa – *Cryptosporidium parvum*, Pfa – *Plasmodium falciparum*, Pbe – *Plasmodium berghei*, Pkn – *Plasmodium knowlesi*, Pvi – *Plasmodium vivax*, Tgo – *Toxoplasma gondii*. **ii) Percentage of similar (upper diagonal) and identical (lower diagonal) amino acids based on optimal global alignment between pairs of sequences showed in iii**. **iii) Multiple sequence alignment of ortholog proteins.** This analyzes show a low conservation along the entire length of representative sequences of eukaryotes, including apicomplexans. Domains are evidenced.

TgRRM_1330

i

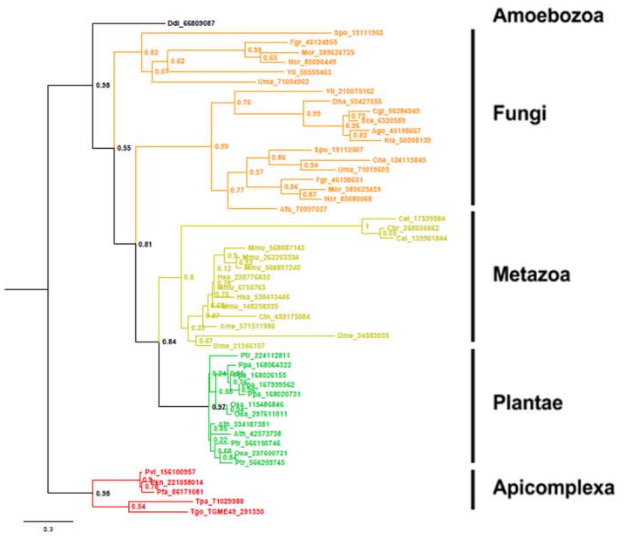

ii

% Similarity (upper diagonal); % Identity (lower diagonal)

	Ddi	Ath	Hsa_AlyRef	Sce_Yra1	Pfa	Tgo	Tcr	Tbr
Ddi	x	21.8	25.8	16.2	24.3	24.2	NC	NC
Ath	15.3	x	55.0	41.9	29.1	37.9	NC	NC
Hsa_AlyRef	18.6	42.5	x	40.6	25.6	35.5	NC	NC
Sce_Yra1	8.4	25.6	25.0	x	24.8	32.9	NC	NC
Pfa	13.8	17.5	13.3	14.0	x	36.1	NC	NC
Tgo	16.1	25.6	24.9	21.5	21.5	x	NC	NC
Tcr	NC	NC	NC	NC	NC	NC	x	NC
Tbr	NC	NC	NC	NC	NC	NC	NC	x

iii

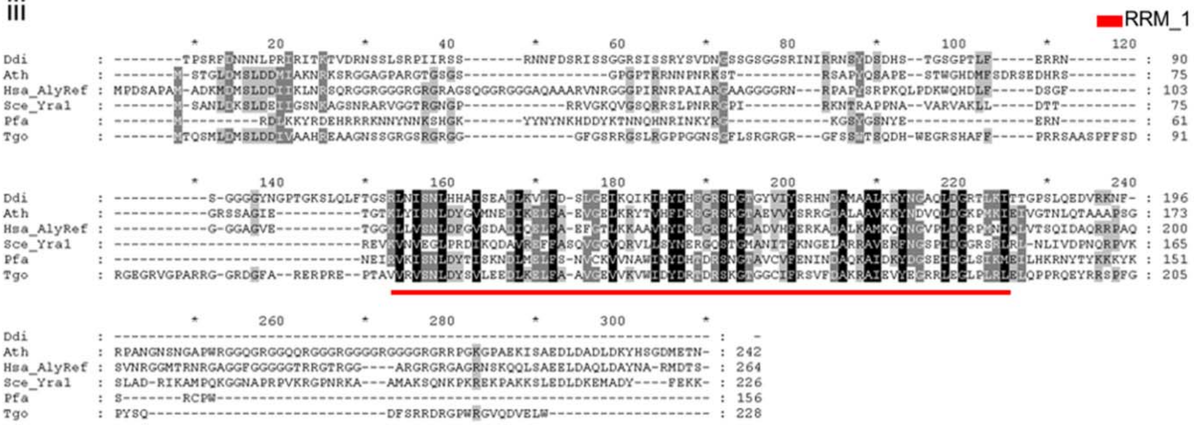

Supp. Figure 6.TgRRM_1330 is less conserved in eukaryotes. A-C. i) Phylogenetic reconstruction of orthologous sequences across different groups of eukaryotes. Numbers at nodes are the bootstrap values. Amoebozoa (black): Ddi – *Dictyostelium discoideum*; Plants (green): Osa – *Oryza sativa*, Ppa – *Physcomitrella patens*, Ath – *Arabidopsis thaliana*, Pop – *Populus trichocarpa*, Olu – *Ostreococcus lucimarinus*, Cre – *Chlamydomonas reinhardtii*, Vca – *Volvox carteri*; Fungi (orange): Uma – *Ustilago maydis*, Cne – *Cryptococcus neoformans*, Spo – *Schizosaccharomyces pombe*, Afu – *Aspergillus fumigatus*, Fgr – *Fusarium graminearum*, Mor – *Magnaporthe oryzae*, Ncr – *Neurospora crassa*, Yli – *Yarrowia lipolytica*, Dha – *Debaryomyces hansenii*, Ago – *Ashbya gossypii*, Kla – *Kluyveromyces lactis*, Cgl – *Candida glabrata*, Sce – *Saccharomyces cerevisiae*; Metazoans (yellow): Cbr – *Caenorhabditis briggsae*, Cel – *Caenorhabditis elegans*, Cin – *Ciona intestinalis*, Hsa – *Homo sapiens*, Mmu – *Mus musculus*, Dme – *Drosophila melanogaster*, Ame – *Apis mellifera*, Mbr – *Monosiga brevicolis*; Parabasilid (blue): Tva – *Trichomonas vaginalis*; Kinetoplastids (purple): Lbr – *Leishmania braziliensis*, Lin – *Leishmania infantum*, Lma – *Leishmania major*, Tbr – *Trypanosoma brucei*, Tcr – *Trypanosoma cruzi;* Apicomplexans (red): Tpa – *Theileria parva*, Cho – *Cryptosporidium hominis*, Cpa – *Cryptosporidium parvum*, Pfa – *Plasmodium falciparum*, Pbe – *Plasmodium berghei*, Pkn – *Plasmodium knowlesi*, Pvi – *Plasmodium vivax*, Tgo – *Toxoplasma gondii*. **ii) Percentage of similar (upper diagonal) and identical (lower diagonal) amino acids based on optimal global alignment between pairs of sequences showed in iii**. **iii) Multiple sequence alignment of ortholog proteins.** This analyzes show a low conservation along the entire length of representative sequences of eukaryotes, including apicomplexans. Domains are evidenced.

-

TgSF2_9530

i

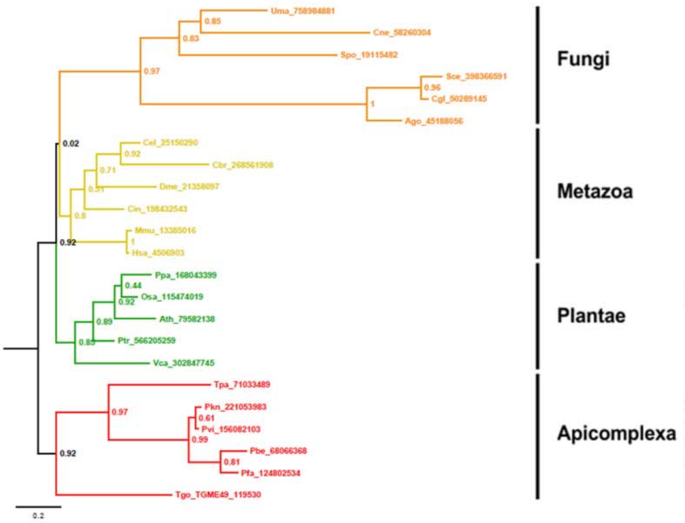

ii

% Similarity (upper diagonal) / % Identity (lower diagonal)

	Ddi	Ath	Hsa	Sce_Npl3	Pfa	Tgo	Tcr	Tbr
Ddi	x	NC	NC	NC	NC	NC	NC	NC
Ath	NC	x	54.5	22.6	27.2	28.8	NC	NC
Hsa	NC	43.3	x	22.6	24.6	26.1	NC	NC
Sce_Npl3	NC	14.1	14.6	x	20	23.5	NC	NC
Pfa	NC	21.5	16.5	12.1	x	41.3	NC	NC
Tgo	NC	22.5	20.5	16.7	28.1	x	NC	NC
Tcr	NC	NC	NC	NC	NC	NC	x	NC
Tbr	NC	NC	NC	NC	NC	NC	NC	x

iii

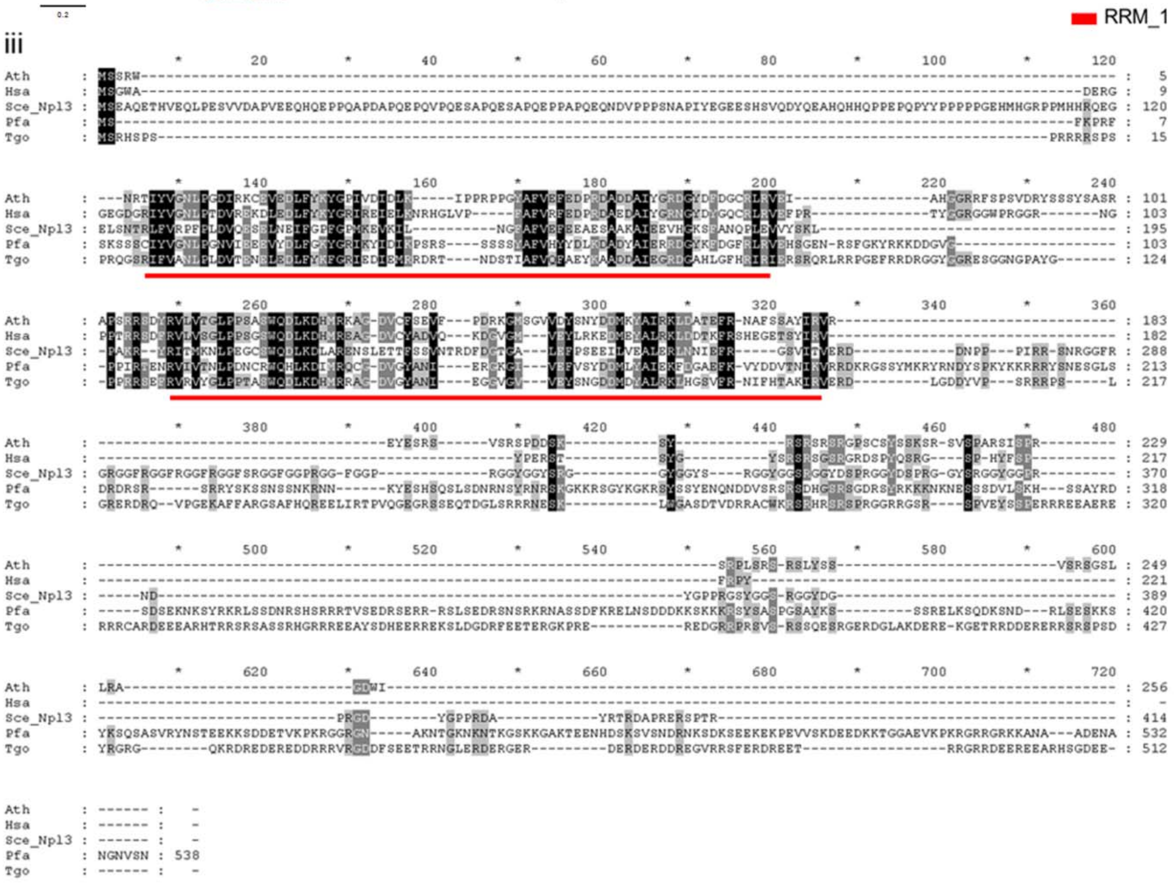

Supp. Figure 7. TgSF2_9530 is less conserved in eukaryotes. A-C. i) Phylogenetic reconstruction of orthologous sequences across different groups of eukaryotes. Numbers at nodes are the bootstrap values. Amoebozoa (black): Ddi – *Dictyostelium discoideum*; Plants (green): Osa – *Oryza sativa*, Ppa – *Physcomitrella patens*, Ath – *Arabidopsis thaliana*, Pop – *Populus trichocarpa*, Olu – *Ostreococcus lucimarinus*, Cre – *Chlamydomonas reinhardtii*, Vca – *Volvox carteri*; Fungi (orange): Uma – *Ustilago maydis*, Cne – *Cryptococcus neoformans*, Spo – *Schizosaccharomyces pombe*, Afu – *Aspergillus fumigatus*, Fgr – *Fusarium graminearum*, Mor – *Magnaporthe oryzae*, Ncr – *Neurospora crassa*, Yli – *Yarrowia lipolytica*, Dha – *Debaryomyces hansenii*, Ago – *Ashbya gossypii*, Kla – *Kluyveromyces lactis*, Cgl – *Candida glabrata*, Sce – *Saccharomyces cerevisiae*; Metazoans (yellow): Cbr – *Caenorhabditis briggsae*, Cel – *Caenorhabditis elegans*, Cin – *Ciona intestinalis*, Hsa – *Homo sapiens*, Mmu – *Mus musculus*, Dme – *Drosophila melanogaster*, Ame – *Apis mellifera*, Mbr – *Monosiga brevicolis*; Parabasilid (blue): Tva – *Trichomonas vaginalis*; Kinetoplastids (purple): Lbr – *Leishmania braziliensis*, Lin – *Leishmania infantum*, Lma – *Leishmania major*, Tbr – *Trypanosoma brucei*, Tcr – *Trypanosoma cruzi;* Apicomplexans (red): Tpa – *Theileria parva*, Cho – *Cryptosporidium hominis*, Cpa – *Cryptosporidium parvum*, Pfa – *Plasmodium falciparum*, Pbe – *Plasmodium berghei*, Pkn – *Plasmodium knowlesi*, Pvi – *Plasmodium vivax*, Tgo – *Toxoplasma gondii*. **ii) Percentage of similar (upper diagonal) and identical (lower diagonal) amino acids based on optimal global alignment between pairs of sequences showed in iii. iii) Multiple sequence alignment of ortholog proteins.** This analyzes show a low conservation along the entire length of representative sequences of eukaryotes, including apicomplexans. Domains are evidenced.

TgRan

i

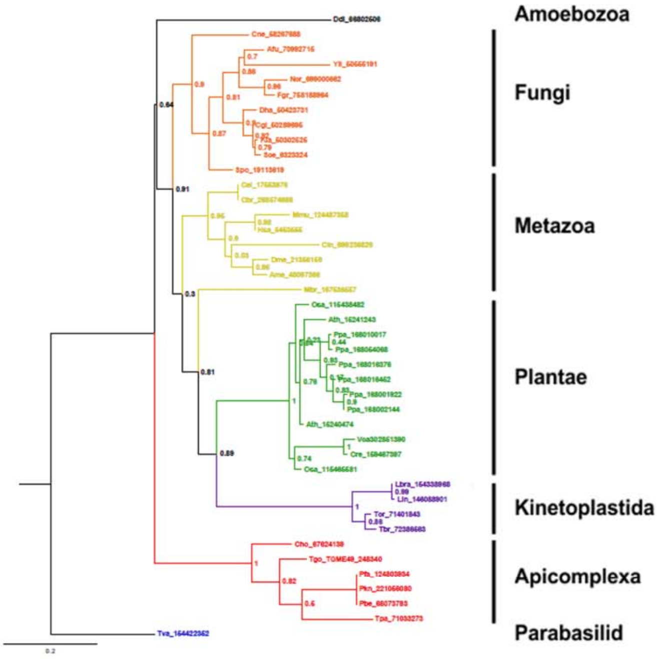

ii

	Ddi	Ath	Hsa_Ran	Sce_Gsp1	Pfa	Tgo	Tcr	Tbr
Ddi	x	74.2	78.7	76.7	77.4	77.0	70.6	68.3
Ath	62.4	x	81.6	84.2	79.2	77.8	74.8	74.7
Hsa_Ran	68.5	72.6	x	86.8	80.6	81.7	76.0	76.0
Sce_Gsp1	68.5	74.3	80.9	x	81.7	83.1	79.0	78.6
Pfa	65.0	64.7	68.1	66.2	x	89.8	74.1	73.7
Tgo	63.6	67.9	70.3	71.2	80.1	x	75.7	75.3
Tcr	60.2	66.5	68.3	68.9	62.0	64.6	x	98.2
Tbr	58.7	66.2	68.3	69.5	60.1	63.4	94.9	x

% Similarity
% Identity

iii

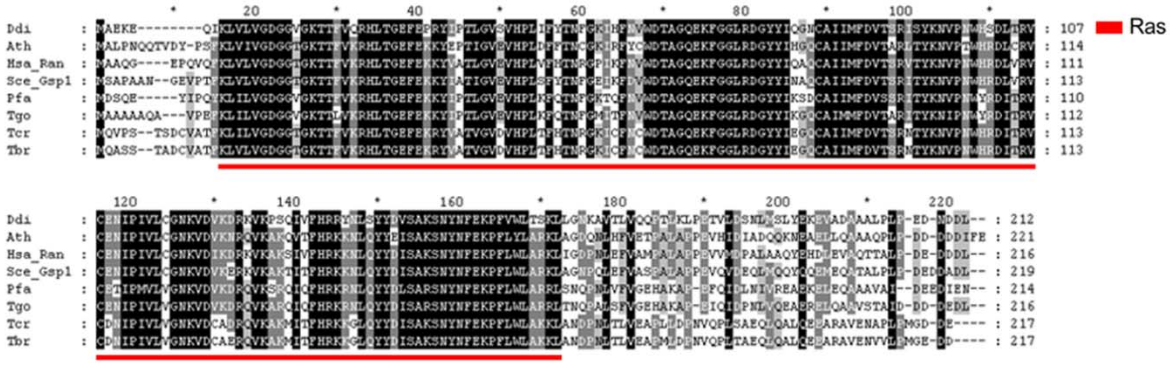

Supp. Figure 8. Ran is highly conserved in eukaryotes. i) Phylogenetic reconstruction of Ran orthologous sequences across different groups of eukaryotes. Numbers at nodes are the bootstrap values. Amoebozoa (black): Ddi – *Dictyostelium discoideum*; Plants (green): Osa – *Oryza sativa*, Ppa – *Physcomitrella patens*, Ath – *Arabidopsis thaliana*, Pop – *Populus trichocarpa*, Olu – *Ostreococcus lucimarinus*, Cre – *Chlamydomonas reinhardtii*, Vca – *Volvox carteri*; Fungi (orange): Uma – *Ustilago maydis*, Cne – *Cryptococcus neoformans*, Spo – *Schizosaccharomyces pombe*, Afu – *Aspergillus fumigatus*, Fgr – *Fusarium graminearum*, Mor – *Magnaporthe oryzae*, Ncr – *Neurospora crassa*, Yli – *Yarrowia lipolytica*, Dha – *Debaryomyces hansenii*, Ago – *Ashbya gossypii*, Kla – *Kluyveromyces lactis*, Cgl – *Candida glabrata*, Sce – *Saccharomyces cerevisiae*; Metazoans (yellow): Cbr – *Caenorhabditis briggsae*, Cel – *Caenorhabditis elegans*, Cin – *Ciona intestinalis*, Hsa – *Homo sapiens*, Mmu – *Mus musculus*, Dme – *Drosophila melanogaster*, Ame – *Apis mellifera*, Mbr – *Monosiga brevicolis*; Parabasilid (blue): Tva – *Trichomonas vaginalis*; Kinetoplastids (purple): Lbr – *Leishmania braziliensis*, Lin – *Leishmania infantum*, Lma – *Leishmania major*, Tbr – *Trypanosoma brucei*, Tcr – *Trypanosoma cruzi;* Apicomplexans (red): Tpa – *Theileria parva*, Cho – *Cryptosporidium hominis*, Cpa – *Cryptosporidium parvum*, Pfa – *Plasmodium falciparum*, Pbe – *Plasmodium berghei*, Pkn – *Plasmodium knowlesi*, Pvi – *Plasmodium vivax*, Tgo – *Toxoplasma gondii*. **ii) Percentage of similar (upper diagonal) and identical (lower diagonal) amino acids based on optimal global alignment between pairs of sequences showed in iii**. **iii) Multiple sequence alignment of Ran ortholog proteins.** This analyzes show a high conservation along the entire length of representative sequences of eukaryotes, including apicomplexans and kinetoplastids. Domains are evidenced.

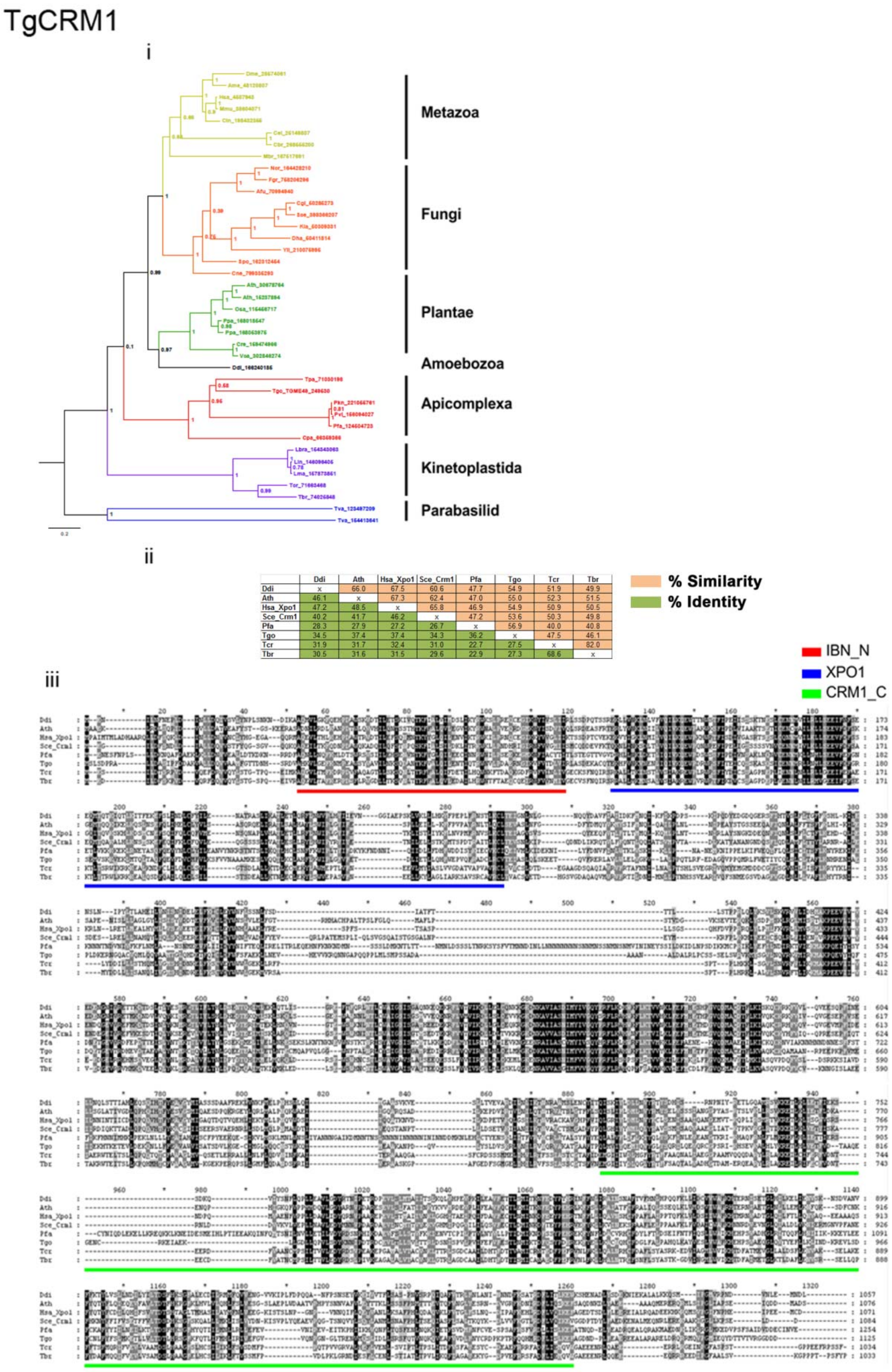

	Ddi	Ath	Hsa_Xpo1	Sce_Crm1	Pfa	Tgo	Tcr	Tbr
Ddi	x	66.0	67.5	60.6	47.7	54.9	51.9	49.9
Ath	46.1	x	67.3	62.4	47.0	55.0	52.3	51.5
Hsa_Xpo1	47.2	48.5	x	65.8	46.9	54.9	50.9	50.5
Sce_Crm1	40.2	41.7	46.2	x	47.2	53.6	50.3	49.8
Pfa	28.3	27.9	27.2	26.7	x	56.9	40.0	40.8
Tgo	34.5	37.4	37.4	34.3	36.2	x	47.5	46.1
Tcr	31.9	31.7	32.4	31.0	22.7	27.5	x	82.0
Tbr	30.5	31.6	31.5	29.6	22.9	27.3	68.6	x

Supp. Figure 9.CRM1 is highly conserved in eukaryotes. i) Phylogenetic reconstruction of CRM1 orthologous sequences across different groups of eukaryotes. Numbers at nodes are the bootstrap values. Amoebozoa (black): Ddi – *Dictyostelium discoideum*; Plants (green): Osa – *Oryza sativa*, Ppa – *Physcomitrella patens*, Ath – *Arabidopsis thaliana*, Pop – *Populus trichocarpa*, Olu – *Ostreococcus lucimarinus*, Cre – *Chlamydomonas reinhardtii*, Vca – *Volvox carteri*; Fungi (orange): Uma – *Ustilago maydis*, Cne – *Cryptococcus neoformans*, Spo – *Schizosaccharomyces pombe*, Afu – *Aspergillus fumigatus*, Fgr – *Fusarium graminearum*, Mor – *Magnaporthe oryzae*, Ncr – *Neurospora crassa*, Yli – *Yarrowia lipolytica*, Dha – *Debaryomyces hansenii*, Ago – *Ashbya gossypii*, Kla – *Kluyveromyces lactis*, Cgl – *Candida glabrata*, Sce – *Saccharomyces cerevisiae*; Metazoans (yellow): Cbr – *Caenorhabditis briggsae*, Cel – *Caenorhabditis elegans*, Cin – *Ciona intestinalis*, Hsa – *Homo sapiens*, Mmu – *Mus musculus*, Dme – *Drosophila melanogaster*, Ame – *Apis mellifera*, Mbr – *Monosiga brevicolis*; Parabasilid (blue): Tva – *Trichomonas vaginalis*; Kinetoplastids (purple): Lbr – *Leishmania braziliensis*, Lin – *Leishmania infantum*, Lma – *Leishmania major*, Tbr – *Trypanosoma brucei*, Tcr – *Trypanosoma cruzi;* Apicomplexans (red): Tpa – *Theileria parva*, Cho – *Cryptosporidium hominis*, Cpa – *Cryptosporidium parvum*, Pfa – *Plasmodium falciparum*, Pbe – *Plasmodium berghei*, Pkn – *Plasmodium knowlesi*, Pvi – *Plasmodium vivax*, Tgo – *Toxoplasma gondii*. **ii) Percentage of similar (upper**

diagonal) and identical (lower diagonal) amino acids based on optimal global alignment between pairs of sequences showed in iii. **iii) Multiple sequence alignment of CRM1 ortholog proteins.** This analyzes show a high conservation along the entire length of representative sequences of eukaryotes, including apicomplexans and kinetoplastids. Domains are evidenced.

Eukaryotic Group	Species	Code	GI
Metazoans	*Homo sapiens*	**Hsa**	4758112
Metazoans	*Mus musculus*	**Mmu**	9790069
Metazoans	*Ciona intestinalis*	**Cin**	198430623
Metazoans	*Drosophila melanogaster*	**Dme**	24581952
Metazoans	*Apis mellifera*	**Ame**	66532824
Metazoans	*Caenorhabditis briggsae*	**Cbr**	157749783
Metazoans	*Caenorhabditis elegans*	**Cel**	71982858
Choanoflagellate	*Monosiga brevicollis*	**Mbr**	167522271
Fungi	*Saccharomyces cerevisiae*	**Sce**	6320119
Fungi	*Candida glabrata*	**Cgl**	50293331
Fungi	*Ashbya gossypiia*	**Ago**	45188133
Fungi	*Kluyveromyces lactis*	**Kla**	50309861
Fungi	*Debaryomyces hansenii*	**Dha**	50424083
Fungi	*Yarrowia lipolytica*	**Yli**	210075100
Fungi	*Magnaporthe oryzae*	**Mor**	145610002
Fungi	*Neurospora crassa*	**Ncr**	164423323
Fungi	*Fusarium graminearum*	**Fgr**	46128571
Fungi	*Aspergillus fumigatus*	**Afu**	70984615
Fungi	*Schizosaccharomyces pombe*	**Spo**	19115173
Fungi	*Cryptococcus neoformans*	**Cne**	58259249
Fungi	*Ustilago maydis*	**Uma**	71021713
Amoebozoa	*Dictyostelium discoideum*	**Ddi**	66826121
Plantae	*Arabidopsis thaliana*	**Ath**	18416493
Plantae	*Oryza sativa*	**Osa**	115437448
Plantae	*Populus trichocarpa*	**Pop**	224092470
Plantae	*Physcomitrella patens*	**Ppa**	168035593
Plantae	*Chlamydomonas reinhardtii*	**Cre**	159491657
Plantae	*Volvox carteri*	**Vca**	302836427
Plantae	*Ostreococcus lucimarinus*	**Olu**	145356615
Apicomplexa	*Theileria parva*	**Tpa**	71027499
Apicomplexa	*Plasmodium berghei*	**Pbe**	68073823
Apicomplexa	*Plasmodium falciparum*	**Pfa**	124801080
Apicomplexa	*Plasmodium knowlesi*	**Pkn**	221053125
Apicomplexa	*Plasmodium vivax*	**Pvi**	156093980
Apicomplexa	*Toxoplasma gondii*	**Tgo**	237843393
Apicomplexa	*Cryptosporidium hominis*	**Cho**	67623541
Apicomplexa	*Cryptosporidium parvum*	**Cpa**	66361130
Kinetoplastida	*Leishmania braziliensis*	**Lbr**	154337278
Kinetoplastida	*Leishmania infantum*	**Lin**	146086104
Kinetoplastida	*Leishmania major*	**Lma**	157869116
Kinetoplastida	*Trypanosoma brucei*	**Tbr**	71746514
Kinetoplastida	*Trypanosoma cruzi*	**Tcr**	71411514
Parabasalid	*Trichomonas vaginalis*	**Tva**	123449042

Supp. Table 1. Genebank Identifier (GI) numbers of UAP56 ortholog proteins identified in 43 representative species of different eukaryotic groups. Amoebozoa: Ddi – *Dictyostelium discoideum*; Plants: Osa – *Oryza sativa*, Ppa – *Physcomitrella patens*, Ath – *Arabidopsis thaliana*, Pop – *Populus trichocarpa*, Olu – *Ostreococcus lucimarinus*, Cre – *Chlamydomonas reinhardtii*, Vca – *Volvox carteri*; Fungi: Uma – *Ustilago maydis*, Cne – *Cryptococcus neoformans*, Spo – *Schizosaccharomyces pombe*, Afu – *Aspergillus fumigatus*, Fgr – *Fusarium graminearum*, Mor – *Magnaporthe oryzae*, Ncr – *Neurospora crassa*, Yli – *Yarrowia lipolytica*, Dha – *Debaryomyces hansenii*, Ago – *Ashbya gossypii*, Kla – *Kluyveromyces lactis*, Cgl – *Candida glabrata*, Sce – *Saccharomyces cerevisiae*; Metazoans: Cbr – *Caenorhabditis briggsae*, Cel – *Caenorhabditis elegans*, Cin – *Ciona intestinalis*, Hsa – *Homo sapiens*, Mmu – *Mus musculus*, Dme – *Drosophila melanogaster*, Ame – *Apis mellifera*, Mbr – *Monosiga brevicolis*; Parabasilid: Tva – *Trichomonas vaginalis*; Apicomplexans: Tpa – *Theileria parva*, Cho – *Cryptosporidium hominis*, Cpa – *Cryptosporidium parvum*, Pfa – *Plasmodium falciparum*, Pbe – *Plasmodium berghei*, Pkn – *Plasmodium knowlesi*, Pvi – *Plasmodium vivax,* Tgo – *Toxoplasma gondii*; Kinetoplastids: Lbr – *Leishmania braziliensis*, Lin – *Leishmania infantum*, Lma - *Leishmania major*, Tcr – *Trypanosoma cruzi*, Tbr – *Trypanosoma brucei.*

Primer	5'- 3'Sequence	final vector	parental parasite strain
uap56 - PstI (F)	CCCCTGCAGGAATGACGACTCTCGAGCAGAACC	ddGFP-UAP56	RH Δ*hxgprt* strain
uap56 - PacI (R)	TTAATTAATCACTGATTGATGTACTGCGAGGCG		
crm1 - XhoI (F)	CCGCTCGAGATGTCGCTTTCAGATCCTCGCGCTCTC	ddGFP-CRM1	RH Δhxgprt strain
crm1 - PacI (R)	CCTTAATTAACTAGTCATCGTCTCCTCCACGAACTGTCA		
TgSF2_9530 - XhoI (F)	CCGCTCGAGATGTCGCGCCACTCTCCCT	ddGFP-NPL3	RH Δhxgprt strain
TgSF2_9530 - PacI (R)	CCTTAATTAATCATTCCTCGTCACCAGAGTGTCTT		
TgRRM_2620 - XhoI (F)	CCGCTCGAGATGCCAGCGGACGAACAGCA	ddGFP-GBP2	RH Δhxgprt strain
TgRRM_2620 - *PacI* (R)	CCTTAATTAACTACGCCACTCGGTCAATTCGC		
ran - PstI (F)	CCCCTGCAGGAATGTCAGATACTGTGTCAGGCC	ddGFP-Ran	RH Δhxgprt strain
ran - PacI (R)	CCCTTAATTAATCACAGGTCCTCATCATCGTCAT		
TgRRM_1330 - PstI (F)	CCCCTGCAGGAATGACACAGTCCATGCTGGACAT	ddGFP-Yra1	RH Δhxgprt strain
TgRRM_1330 - PacI (R)	CCCTTAATTAATCACCATAGTTCGACGTCTTGAA		
TgU2_6910 - PstI (F)	CCCCTGCAGGAATGGCGGAGCATCTGGCTCGTAT	ddGFP-U2AF35	RH Δhxgprt strain
TgU2_6910 - PacI (R)	CCCTTAATTAATCATCCATTCGCTTCTTCGCG		
5UTR-*KpnI* (F)	GGGGTACCCTGCTTGAGGTCTTGCTTGC	*loxPUap56loxP-mCherry-HX*	RH DiCre DKu80 strain
5UTR-*loxP*-*ApaI* (R)	GGGGGCCCATAACTTCGTATAGCATACATTATACGAAGTTATTTGTCAACGCGGTTGAGGG		
uap56-ApaI (F)	GGGGGCCCATGACGACTCTCGAGCAGAAC	*loxPUap56loxP-mCherry-HX*	RH DiCre DKu80 strain
uap56-loxP-NsiI (R)	GCTAATGCATTTTATAACTTCGTATAGCATACATTATACGAAGTTATTTACTGATTGATGTACTGCGAGG		
3UTR-*SacI* (F)	GGGGAGCTCAGTCGAACACAGTAAACGTTTCTTG	*loxPUap56loxP-mCherry-HX*	RH DiCre DKu80 strain
3UTR-*SacI* (R)	GGGGAGCTCCCTAGGAAGGCGTCGTGGATCCTTATT		
I-5UTR ext F (external)	GTATTAGTACATGGTGTTCAAGCG		
II-UAP56 int R (internal)	CACTTGTGTCCAGGTTCAGCT		
III-UAP56 F	ATGACGACTCTCGAGCAGAAC		
IV-mcherry R (internal)	CACCGGCGGCATGGACGAGCTGTACAAG		
V-mcherry F	ATGCTATACGAAGTTATAAAATGCA		
VI-3UTR R ext (external)	CATGTCGGGTTAGTTCATATCGT		
VII-HX F	GCTCAATACGGCCCTTGCCCTTGC		
gap40 (F)	CCGCAATTGAGATCTATGTCGACTCTTCAGGACATTCGCTTG		
gap40 (R)	GGGTTAATTAATGCATTAGCTCGAATGGGCTTCGTCGTCAC		
PolII p8.2 (F)	ATGATCATCCCAATTCGGTGCT		
PolII p8.2 (R)	TCAGCTCTCCACAATGCGCTT		
PolII p19 (F)	GTCCTTGACGCCGTCATCACC		
PolII p19 (R)	TCAAAGCGTCGGAGAATGGGG		
PolII p23(F)	GTCAAAGAGATTCAGCTTCCAAGGATTC		
PolII p23 (R)	GTCACGAGAACTTGCAGTACACG		
IMC1 (F)	GAGGTACCTGAGGTTCAGTACGTC		
IMC1 (R)	AAGACCTGCTCCACCTTGGG		
IMC15 (F)	GTTCCAAAGGAATTCACGCGCATTG		
IMC15 (R)	CTGTTAGCCGTTCTTTATCTAGGTGAGTG		
IMC5 (F)	CTGTTAGCCGTTCTTTATCTAGGTGAGTG		
IMC5 (R)	CTCCATTTCGACAGTCAAGAGTCCAG		
TFIID (F)	ATGACAACTGAGGACGACGAGACA		
TFIID (R)	CTACTGATGGTAGCGAACAAGCACG		
T7	TGTAATACGACTCACTATAGGGC		
Sp6	ATTTAGGTGACACTATAGAATACTC		

list of gRNA sequences					final vector
Gene	ToxoID	Start position	gRNA sequence	PAM	
GAP40	TGME49_249850	553	TGGAGTACAGCACTGCCCAT	GGG	pU6-gRNA-crisprRNAs GAP40
UAP56.1	TGME49_216860	3186	ATCAGCAGTTCAAGAACTTC	GAG	pU6-gRNA-crisprRNAs UAP56
UAP56.2	TGME49_216860	2287	GGACGAGTGCGACAAGTGCT	TGG	
CRM1.1	TGME49_249530	123	CGTCGCACATAAAGTTCTGG	GAG	pU6-gRNA-crisprRNAs CRM1
CRM1.2	TGME49_249530	234	GATTCTTGAAAACACGATCC	AGA	
TgRRM_1330	TGME49_091330	1688	AGGCAGAGACGGTTTTGCGA	GGG	pU6-gRNA-crisprRNAs TgRRM_1330
RAN	TGME49_248340	489	GAGTTCAAACTTATCCTCGT	CGG	pU6-gRNA-crisprRNAs RAN
TgSF2_9530	TGME49_119530	63	GGAGGTTGGCAACGAAGATT	CGG	pU6-gRNA-crisprRNAs TgSF2_9530
TgRRM_2620	TGME49_062620	538	AACAGCAACAGCAACTCCCG	CGG	pU6-gRNA-crisprRNAs TgRRM_2620
TgU2_6910	TGME49_236910	12	TCTGGCTCGTATCATAGGGA	CGG	pU6-gRNA-crisprRNAs TgU2_6910
LacZ	-	303	GCTGGAGTGCGATCTTCCTG	AGG	pU6-gRNA-crisprRNAs LACZ

Supp. Table 2. Oligonucleotides used in this study. Name and sequence of individual oligonucleotides are listed. The restriction enzymes used are in the name of primer and its respective recognized sequence in italic. *loxP* sequences added to reverse primers are underlined.
